# A comprehensive review of in planta stable transformation strategies

**DOI:** 10.1186/s13007-024-01200-8

**Published:** 2024-05-31

**Authors:** Jérôme Gélinas Bélanger, Tanya Rose Copley, Valerio Hoyos-Villegas, Jean-Benoit Charron, Louise O’Donoughue

**Affiliations:** 1 Centre de recherche sur les grains (CÉROM) Inc., 740 Chemin Trudeau, St-Mathieu-de-Beloeil, Québec J3G 0E2 Canada; 2 Department of Plant Science, McGill University, 21111 Lakeshore Road, St-Mathieu-de-Beloeil, Montréal, Québec H9X 3V9 Canada

**Keywords:** In planta transformation, In situ transformation, Direct organogenesis, Indirect organogenesis, Recalcitrant species, In vivo regeneration

## Abstract

Plant transformation remains a major bottleneck to the improvement of plant science, both on fundamental and practical levels. The recalcitrant nature of most commercial and minor crops to genetic transformation slows scientific progress for a large range of crops that are essential for food security on a global scale. Over the years, novel stable transformation strategies loosely grouped under the term “in planta” have been proposed and validated in a large number of model (e.g. *Arabidopsis* and rice), major (e.g. wheat and soybean) and minor (e.g. chickpea and lablab bean) species. The in planta approach is revolutionary as it is considered genotype-independent, technically simple (i.e. devoid of or with minimal tissue culture steps), affordable, and easy to implement in a broad range of experimental settings. In this article, we reviewed and categorized over 300 research articles, patents, theses, and videos demonstrating the applicability of different in planta transformation strategies in 105 different genera across 139 plant species. To support this review process, we propose a classification system for the in planta techniques based on five categories and a new nomenclature for more than 30 different in planta techniques. In complement to this, we clarified some grey areas regarding the in planta conceptual framework and provided insights regarding the past, current, and future scientific impacts of these techniques. To support the diffusion of this concept across the community, this review article will serve as an introductory point for an online compendium about in planta transformation strategies that will be available to all scientists. By expanding our knowledge about in planta transformation, we can find innovative approaches to unlock the full potential of plants, support the growth of scientific knowledge, and stimulate an equitable development of plant research in all countries and institutions.

**Supplementary Information:**

The online version contains supplementary material available at 10.1186/s13007-024-01200-8.

## Introduction

Although it has been more than 40 years since the first publications concerning transgenic plants, plant transformation remains a major bottleneck in most commercially important and underutilized crops [1]. The recalcitrant nature of many plant species and genotypes to in vitro regeneration is a significant barrier to plant improvement, thus slowing scientific progress and contributing to an overreliance on the same species and genotypes that are more easily amenable to transformation. However, several transformation strategies devoid of or with minimal tissue culture steps have been developed over the years. Altogether these methods offer a promising alternative to the laborious tissue culture steps associated with in vitro techniques. Such transformation strategies are loosely termed “in planta” and have been proven efficient in a breadth of monocot and dicot species. Generally, most in planta methods are also often considered genotype-independent since they do not rely heavily on hormone supplementation and often omit the callus regeneration step. As such, in planta strategies are less prone to somaclonal variations and offer an alternative to circumvent the challenges associated with these long-lasting genetic changes. The simple and affordable nature of these protocols in comparison to in vitro methods makes them particularly suited for minor crops. This feature can allow labs to manage simultaneous genetic transformation projects using various species, genotypes, and constructs with minimal financial requirements and trained personnel. On a global level, these aspects can guarantee an equitable development of plant research in all countries, institutions, and budgets. Moreover, the negligible financial inputs required by labs to undergo in planta projects signifies that riskier projects can be undertaken.

To this day, the only in planta method that has received widespread attention is the *Arabidopsis thaliana* floral dip method. The floral dip method is one of the most cited protocols in plant molecular biology and is one of the main factors that has contributed to propelling *Arabidopsis* to the honorable status of “most important model organism in plant biology” [2–4]. As a whole, the success of this technique clearly depicts the potential of development for universal in planta methods, particularly in the era of CRISPR-Cas9 and high-throughput genome editing. Over the years, several review papers have been written on the topic of in planta transformation, thus demonstrating the importance of the concept [5–11]. Largely, papers focused on specific in planta methods, such as the floral dip and the shoot apical meristem (SAM) injury techniques, and do not include the most recent scientific developments in an area that is rapidly evolving. This article aims at complementing these past literature reviews and framing them into the bigger context of in planta transformation as a topic. Overall, we start this review by drawing the conceptual framework of in planta stable transformation and classifying the different in planta strategies. Subsequently, we describe several in planta experimental approaches with a focus on recent advances and finally discuss the future avenues and possibilities in this field of research.

## Approaches for data collection and building of the in planta compendium

**Fig. 1:**
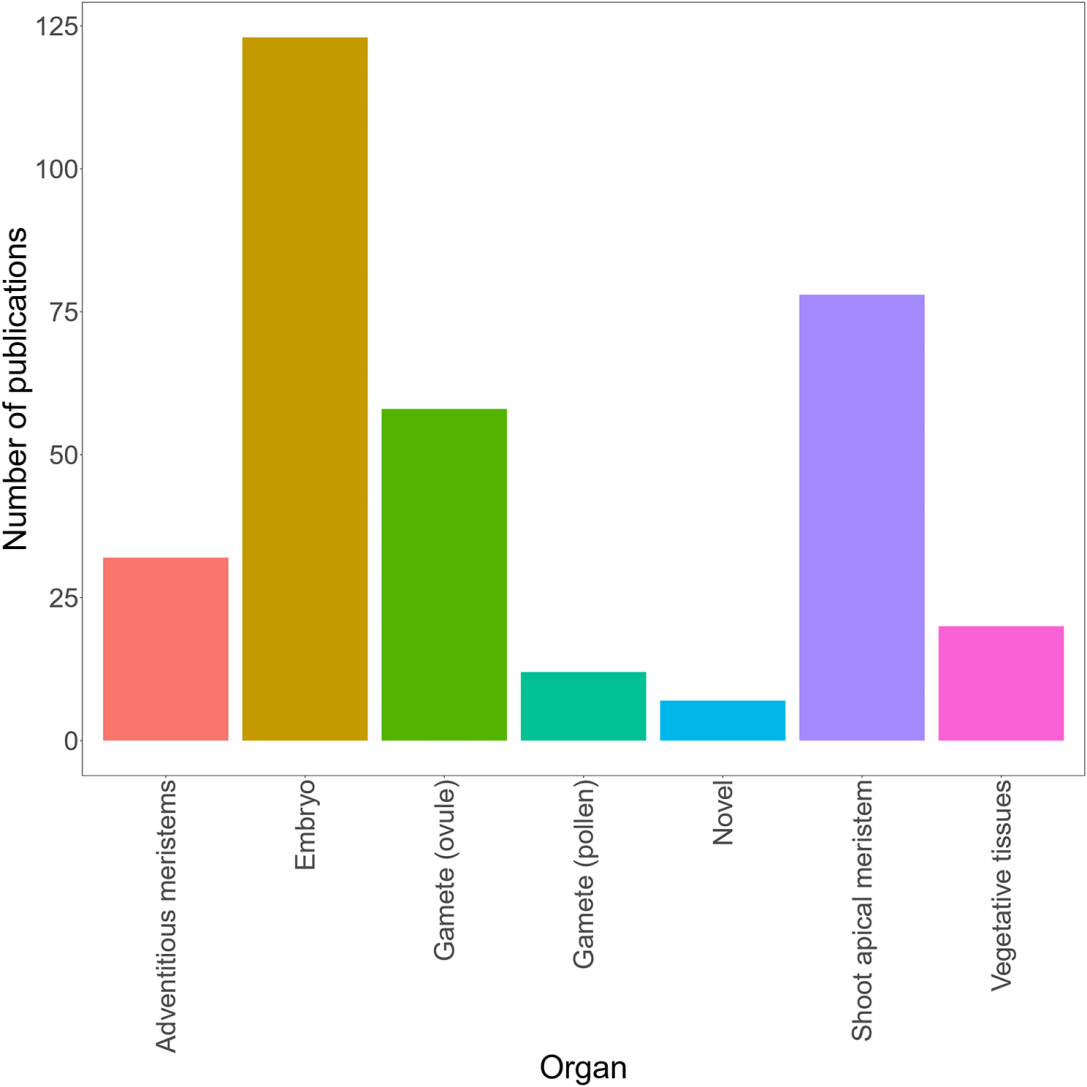
Distribution of the publications found in the in planta compendium. This graph shows the distribution of the publications associated with each type of explant

**Fig. 2:**
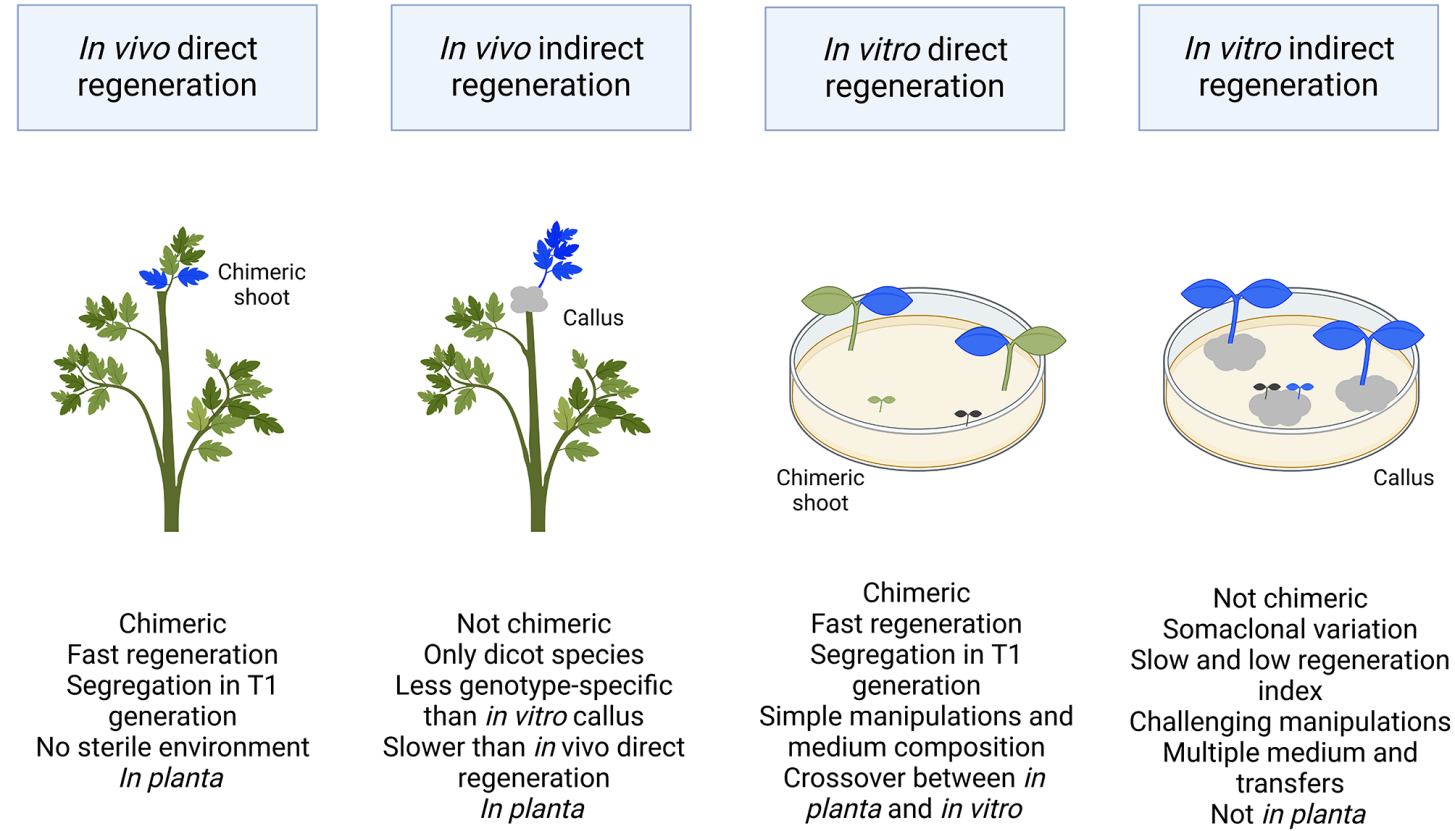
Classification of the four de novo organogenesis pathways. Regeneration-dependent de novo organogenesis strategies can be performed under in vivo (in planta) or in vitro (not in planta) conditions. The direct regeneration mechanism has many advantages over the indirect mechanism as it is simpler and quicker to perform; however, it leads to the formation of chimeric T_0_ mutants that require segregation in the T_1_ generation to obtain non-chimeric offspring. Moreover, the direct regeneration mechanism does not suffer from somaclonal variation, unlike callus-based methods. Callus-based methods are generally more challenging to perform but can be useful for specific crops (e.g. plants with a long juvenile phase such as trees) that cannot be transformed efficiently using the direct regeneration mechanism. The in vitro indirect regeneration pathway is generally considered highly genotype-dependent due to the use of multiple growing media, whereas direct regeneration methods are more universal due to their use of simple cultivation medium that are suitable for a larger spectrum of genotypes. The classification of these pathways was inspired by the comparative scheme of bud regeneration avenues developed by Shi et al. [54]

**Fig. 3:**
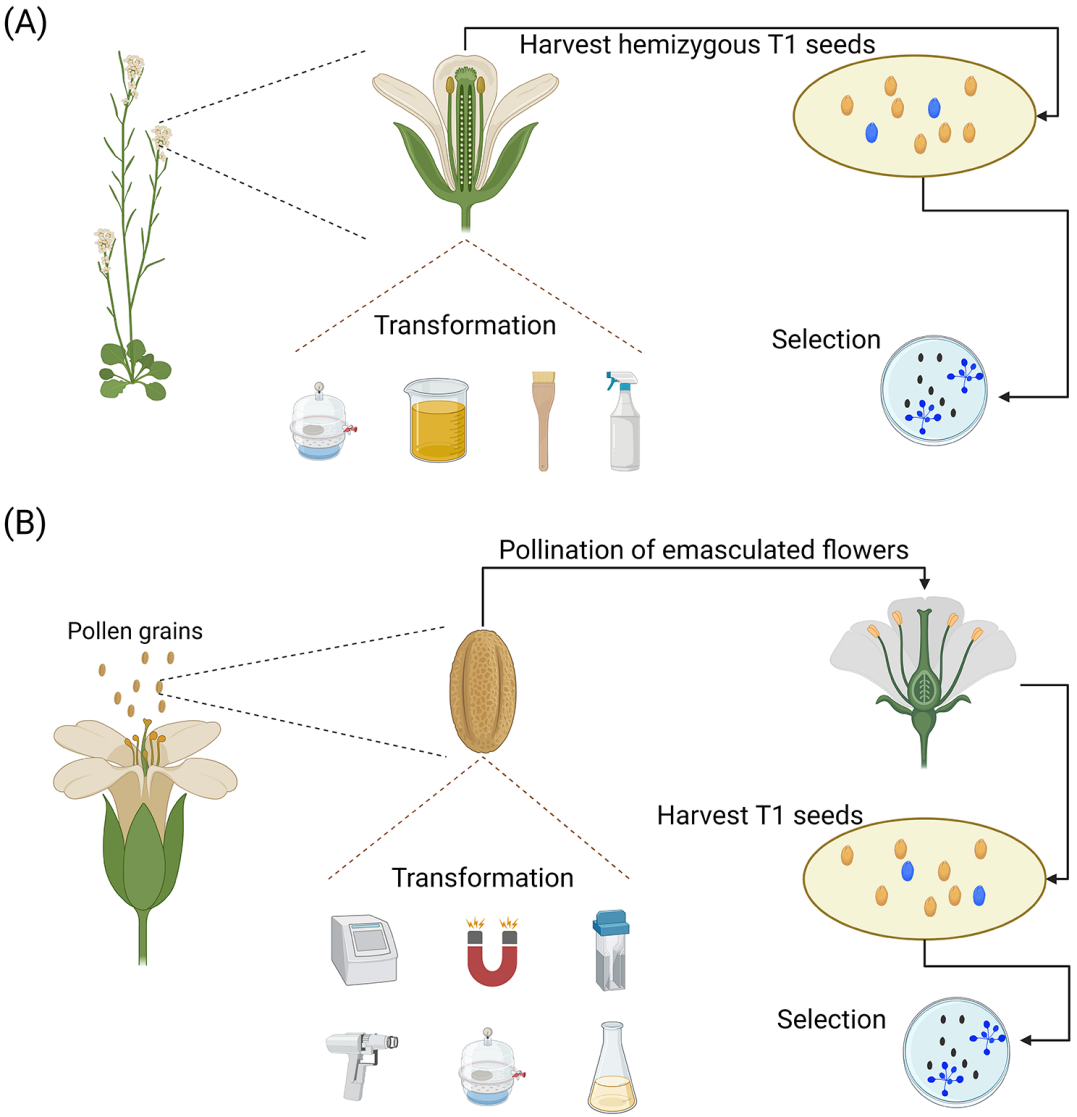
Gamete-based transformation techniques. (**A**) Strategies targeting the female gamete (ovule). Several in planta techniques (e.g. the floral dip [59], vacuum-infiltration [60], floral spray [76], and floral painting [67]) targeting the female gametes have been developed and validated. In *Arabidopsis*, in planta strategies targeting the ovules often lead to the generation of hemizygous offspring in the T_1_ generation as the male reproductive organs (i.e. pollen and pollen tubes) remain untouched [137, 243]. A thorough screening must be performed in the T_1_ generation and further to identify positive mutants using a selection marker or reporter gene [65, 66]. (**B**) Male gametes-based in planta approaches. In these strategies, the pollen grains are transformed through various methods such as sonication [83], vacuum infiltration [82], magnetofection [85, 86], *Agrobacterium* [82, 84], particle bombardment [80, 81], and electroporation [79]. Subsequently, these pollen grains are used to pollinate the recipient plant’s ovules and lead to the generation of putatively transformed T_1_ offspring. Following this, screening is performed in the T_1_ generation to identify positive transformants

**Fig. 4:**
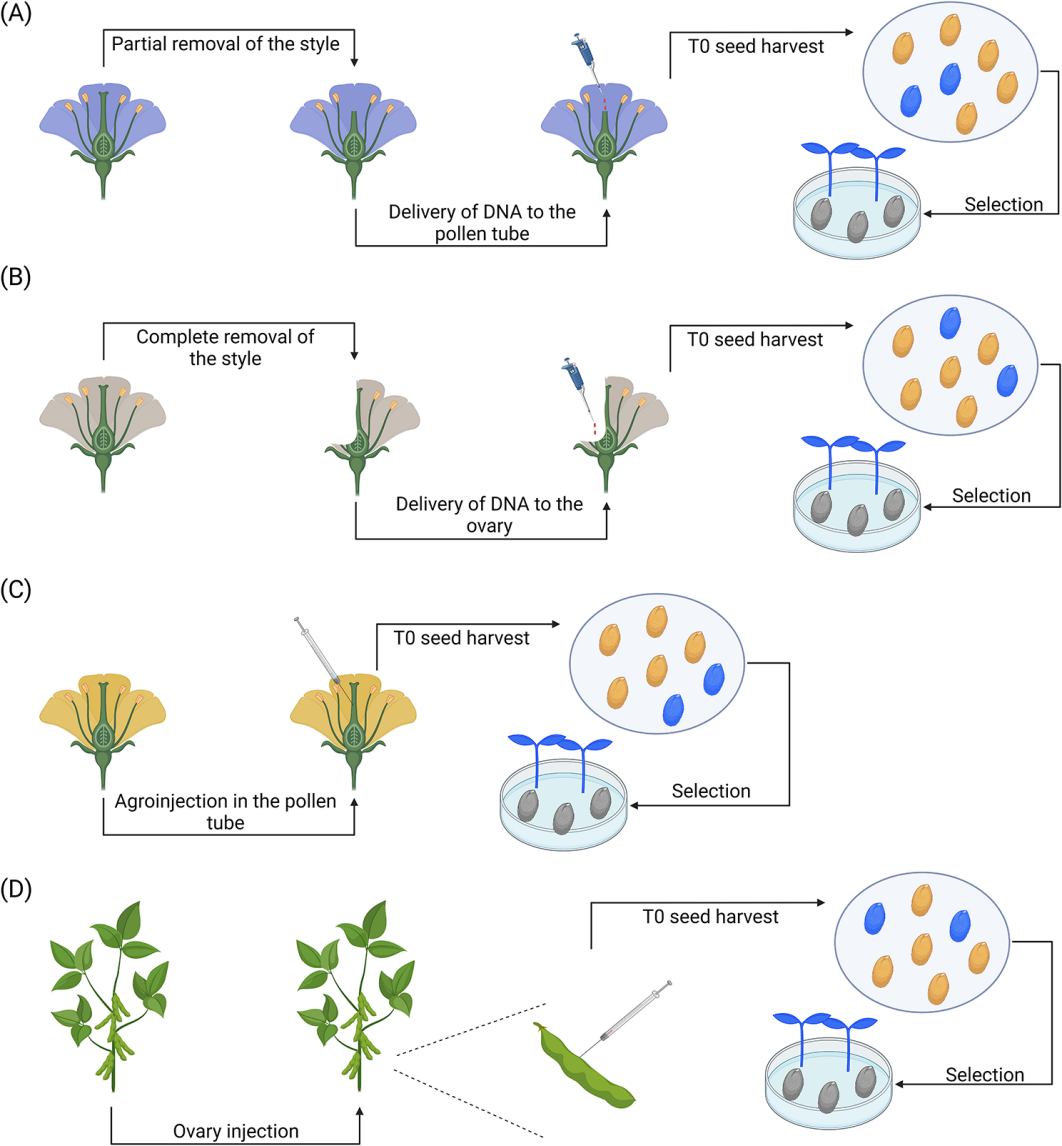
In planta approaches targeting the embryos at an early stage of development. (**A**) Pollen-tube pathway [92]. To perform the pollen-tube pathway, the plant’s stigmas are removed and the styles are severed shortly after pollination. Subsequently, exogenous donor DNA is applied to the severed styles and delivered to the recipient plant’s ovaries via the growth of the pollen tube. Following the seed set, the putative transformants are screened to identify positive mutants. (**B**) Ovary-drip [92]. In this approach, the ovary sac is incised using a sterile scalpel, and exogenous DNA is directly delivered to the ovule drop-by-drop using a micropipette. (**C**) Pollen-tube agroinjection [113]. In this method, a solution of resuspended *Agrobacterium* is injected into the plant’s pollen tube using injector needles. To do so, the carina is punctured with the needles and the solution is injected until the wing petals are soaked. (**D**) Ovary injection [115, 116, 118]. To apply the ovary injection strategy, a solution of resuspended *Agrobacterium* is injected into the ovaries (i.e. soybean pods in this case) at an early stage of development to infect the developing embryos. Following this step, the mature seeds are further screened to identify positive mutants

**Fig. 5:**
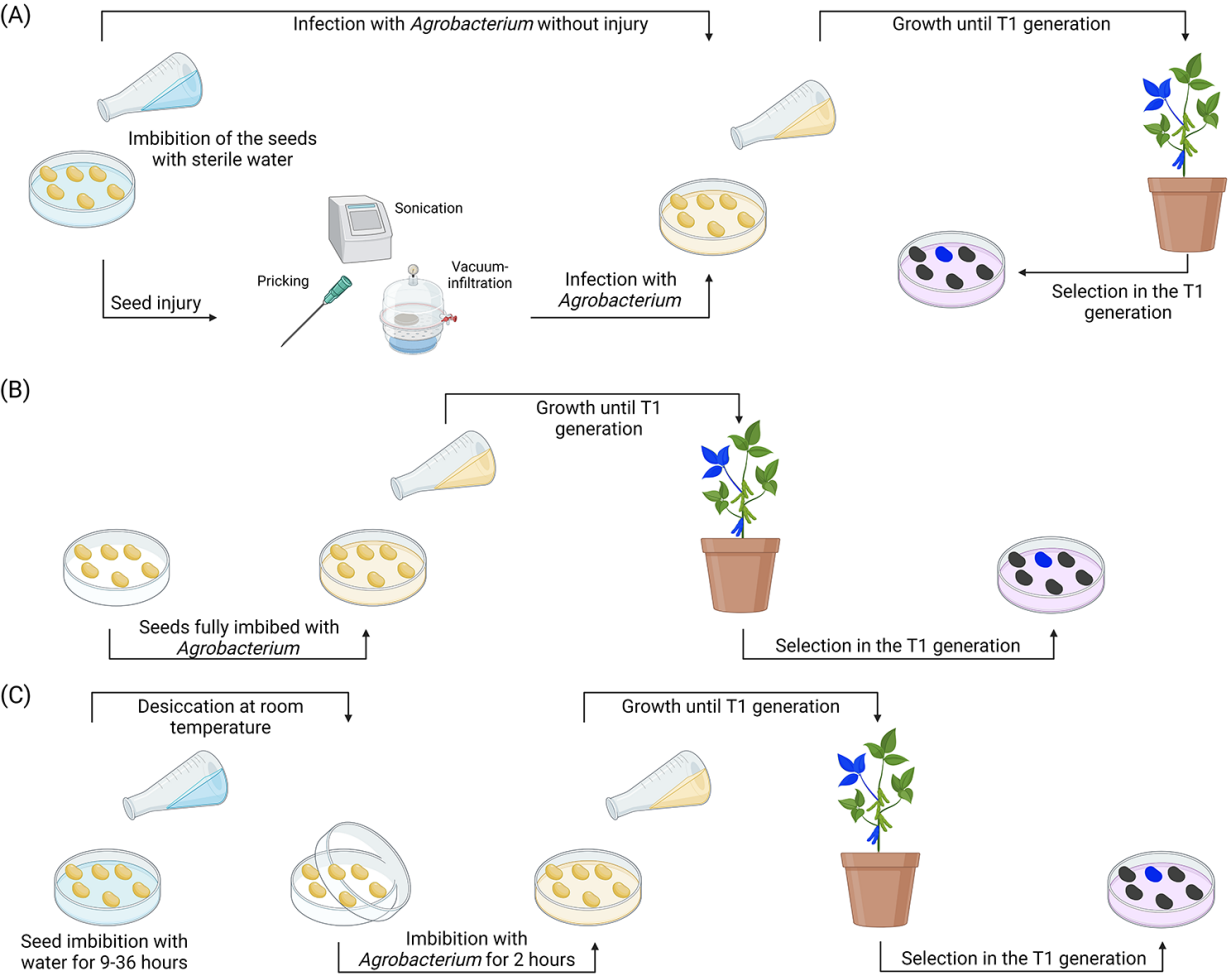
In planta strategies targeting the embryos at a later stage of development. (**A**) Infection of pre-imbibed embryos with *Agrobacterium*. The seeds are imbibed with sterile water and either (i) kept uninjured [122] or (ii) injured using pricking, sonication, or vacuum infiltration [121]. Following this treatment, the seeds are infected with a solution of *Agrobacterium* and grown until the T_1_ generation for selection. (**B**) Agro-imbibition [124]. In this approach, seeds are imbibed with a solution of *Agrobacterium* instead of sterile water and further selected in the T1 generation. (**C**) Imbibition of desiccated embryos [125]. To perform this method, seeds are first imbibed with sterile water and subsequently desiccated at room temperature for 9–36 h. The seeds are subsequently infected for 2 h with a solution of *Agrobacterium* and cultivated until the T_1_ generation for selection

**Fig. 6:**
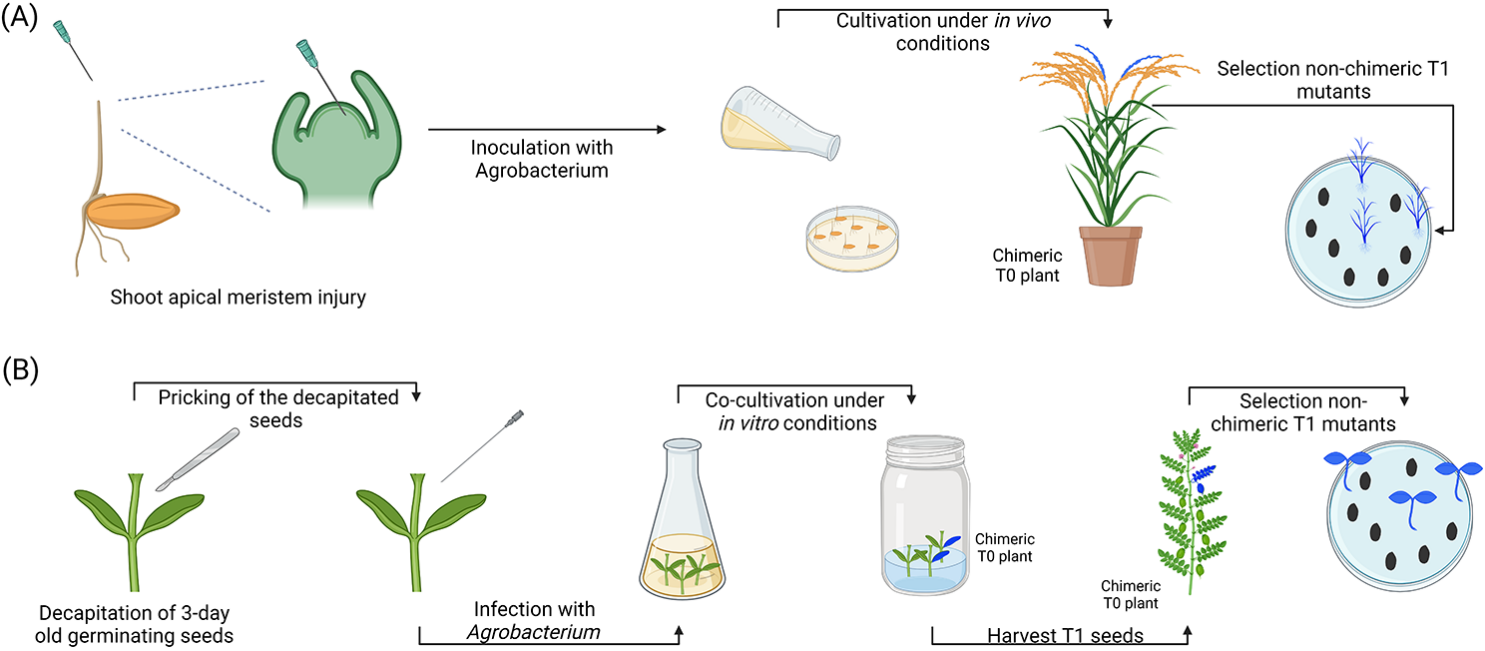
Transformation approaches targeting the apical and adventitious meristems. (**A**) Shoot apical meristem injury under in vivo conditions [129]. The apical meristematic region is pricked with a needle and subsequently infected with resuspended *Agrobacterium*. Chimeric T_0_ plants are grown under in vivo conditions until seed set. Non-chimeric lines are further selected in the T_1_ generation. (**B**) Plumular meristem approach [22, 148]. In the plumular meristem approach, young seedlings are decapitated and their radicules excised with a sterile scalpel. Following this treatment, the explants are infected with *Agrobacterium* and co-cultivated on a sterile medium under in vitro conditions. After co-cultivation, the seedlings are moved to greenhouse conditions and allowed to set seeds. The T_1_ offspring are then screened to identify positive mutants

**Fig. 7:**
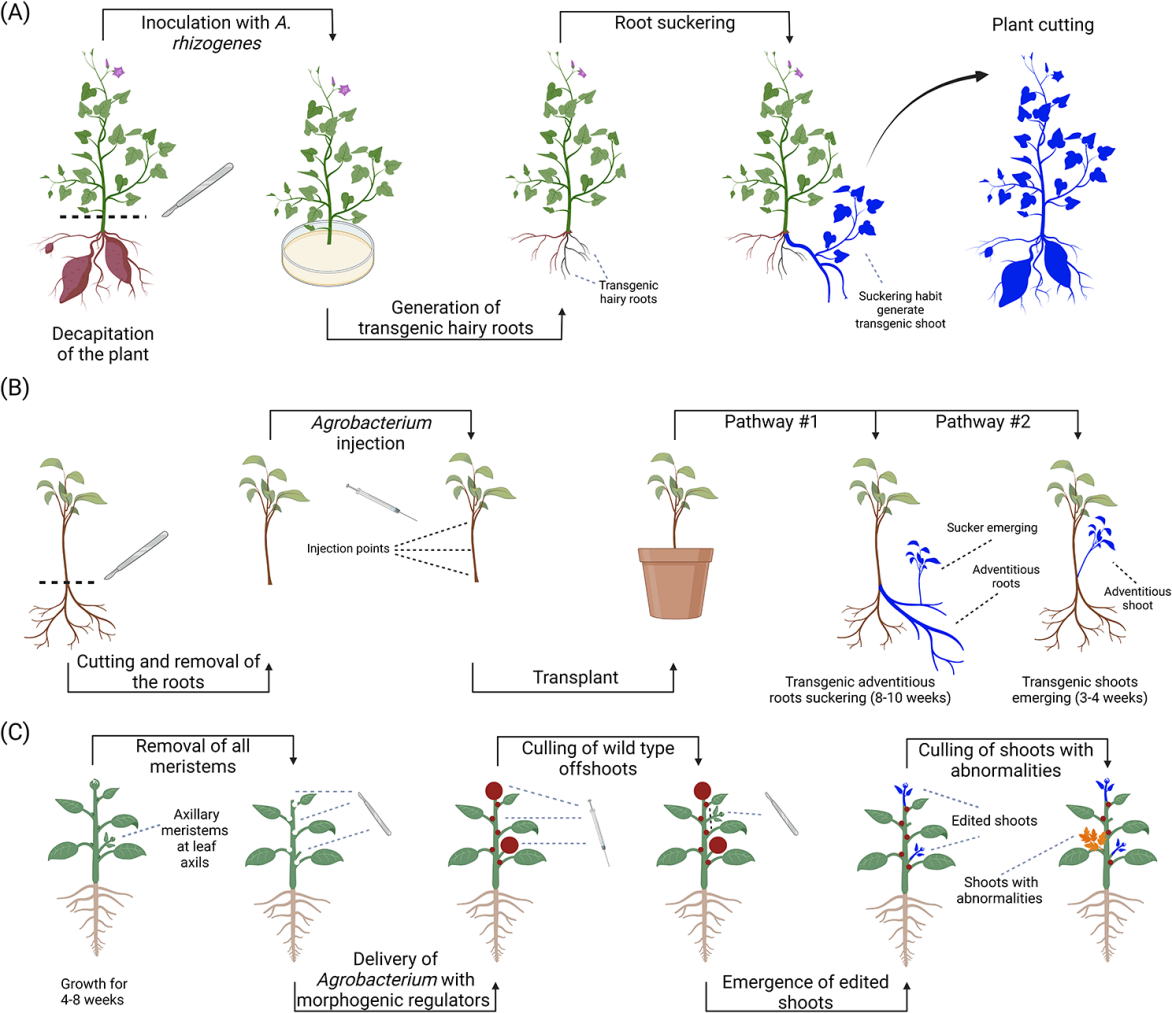
Additional in planta techniques targeting the shoot apical and adventitious meristems. (**A**) Direct organogenesis of propagules (cut-dip-budding technique) [41]. To perform this method, plants with a high asexual reproduction capacity (e.g. sweet potato) are decapitated and their wounds are treated with a solution of resuspended *Agrobacterium rhizogenes*. Due to the root-suckering features of these plants, transgenic hairy roots will slowly develop and generate a newly transformed plant. (**B**) Direct organogenesis of propagules (Regenerative activity-dependent in planta injection delivery technique) [150]. In the RAPID method, a solution of resuspended *A. tumefaciens* is injected into the stem of plants with a high asexual reproduction capacity such as sweet potato. The plant is subsequently transplanted and transformed roots (pathway #1) or shoots (pathway #2) will subsequently emerge from the wound sites. (**C**) Direct delivery of exogenous morphogenic regulators [175, 244]. In the Direct delivery approach, the recipient plants’ meristems are removed using a sterile scalpel, and developmental regulators (e.g. *WUSCHEL/WUSCHEL2*) are subsequently delivered by injecting a solution of resuspended *A. tumefaciens* into the wound sites. Following this, the wild-type abnormal transgenic offshoots are culled, whereas the normal transgenic shoots are identified for further propagation

**Fig. 8:**
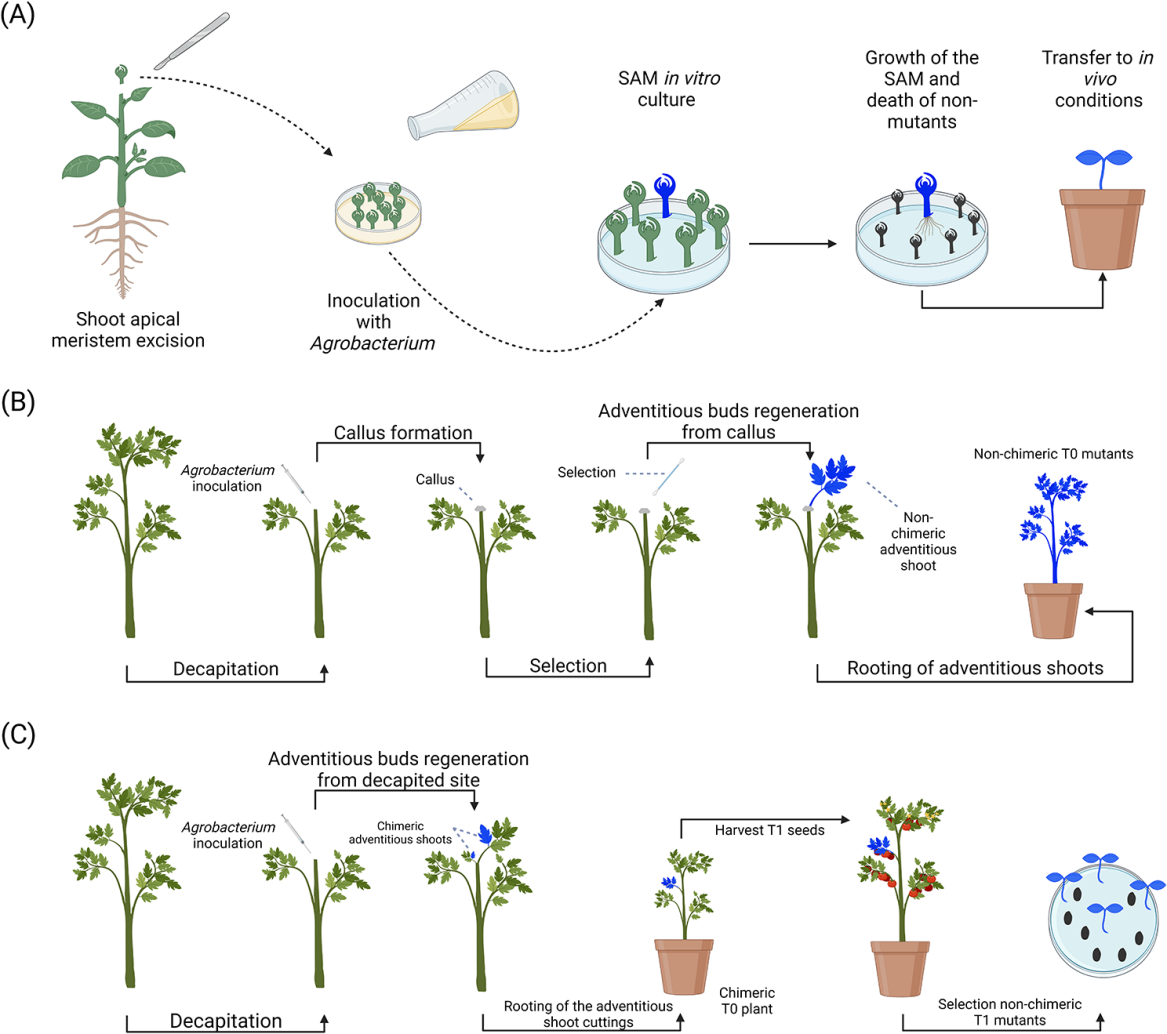
In planta transformation using in vitro direct organogenesis and in vivo callus-based approaches. (**A**) In vitro direct organogenesis. The shoot apical meristems (SAM) are excised from the growing seedlings and inoculated with resuspended *Agrobacterium* [57, 58]. Following inoculation, the putatively transformed shoot apical meristems are grown and screened under in vitro conditions to identify positive T_0_ mutants. Following the screening process, mutants are rooted and then transferred to in vivo conditions for seed setting. Optionally, embryonic axes from imbibed seeds can be used similarly to shoot apical meristems (details not shown in the figure) [55, 56, 245]. (**B**) In vivo callus regeneration [16, 17, 19, 210]. Dicot plants are decapitated and their wound sites are injected or rubbed with a solution of *Agrobacterium*. Subsequently, the wound sites are covered with parafilm and/or aluminum foil to retain moisture and keep the sites under dark conditions to favor callus formation. Optionally, the wounds can be treated with different hormones to promote the formation of a callus. Before or after callus formation, the sites can be treated with a selection marker such as an antibiotic or herbicide to eliminate untransformed calli cells. After the callus is formed, shoot formation is favored by cultivating the callus site under a regular photoperiodic regime. Under these conditions, transformed shoots will emerge from the calli cells surviving the screening process. (**C**) Shoot apical meristem removal and direct regeneration of adventitious meristems [16, 19, 210]. Plants are decapitated and the wound site is inoculated with *Agrobacterium* through injection and/or rubbing. The wound site is subsequently covered with parafilm and/or aluminum foil to retain moisture and keep it under dark conditions. Chimeric plants regenerate from the wound site and the adventitious shoot can be maintained on the same plant, grafted on another plant, or rooted in a separate container. Selection is performed in the T_1_ generation to retrieve non-chimeric offspring

**Fig. 9:**
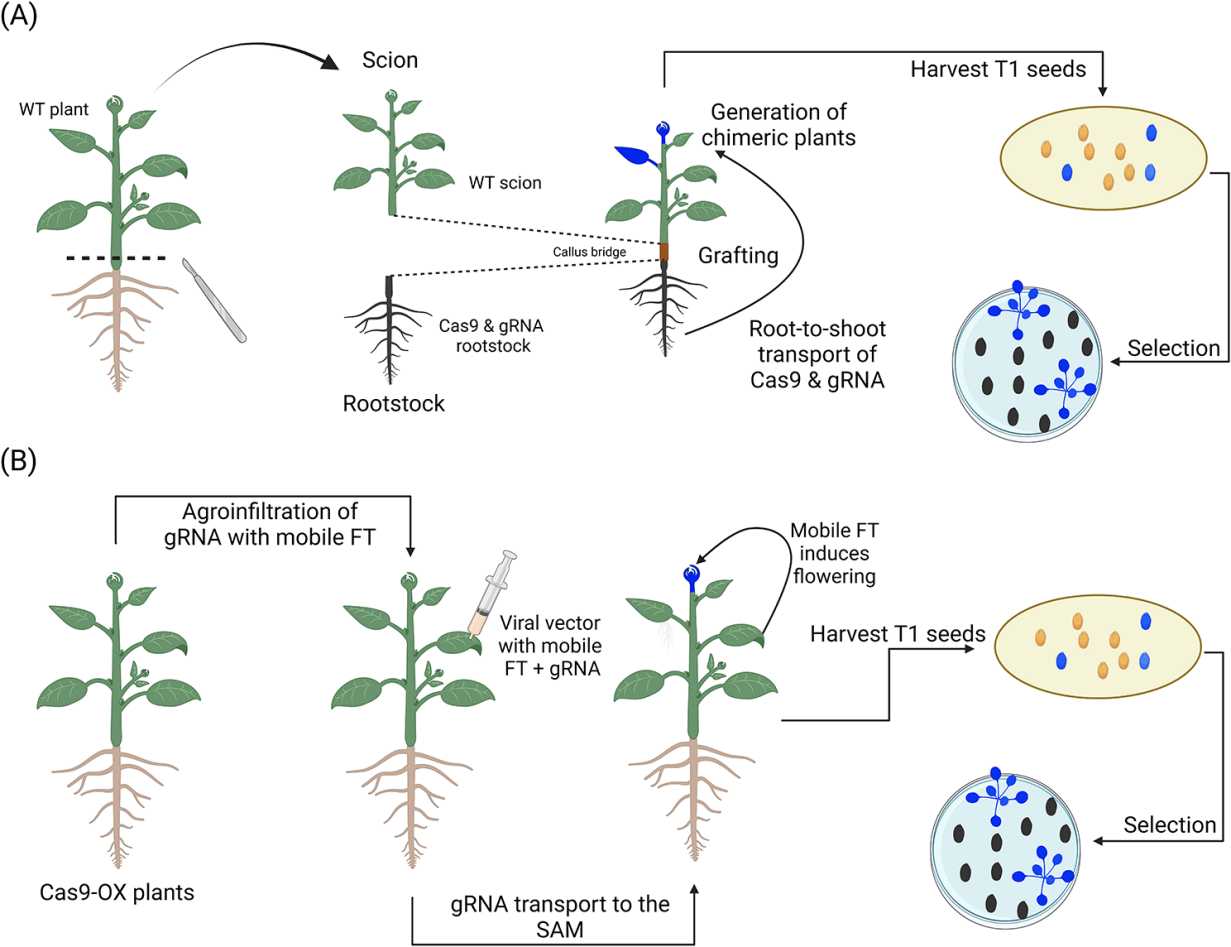
Novel transformation techniques used for in planta transformation. (**A**) Grafting-mediated transformation [227]. Wild-type scion is grafted to a transgenic rootstock containing Cas9 and gRNA sequences. The grafting procedure leads to the formation of chimeric scions containing Cas9 and gRNA sequences due to the movement of tRNA-like sequence motifs that ensure transcript mobility across the plant. The rootstock to scion movement of these sequences causes heritable edits in the germline cells and edited offspring can be retrieved upon selection in the T_1_ generation. (**B**) Viral-based vector using a mobile *FT* cassette [238]. The leaves of mutant plants overexpressing Cas9 are agroinfiltrated with a viral vector (e.g. tobacco rattle virus vector) containing a gRNA sequence fused to mobile *FT* sequences. The gRNA sequence reach the germline cells of the Cas9 overexpressing mutants upon the transcription of *FT* due to its endogenous natural movement to the shoot apical meristem and the edited offspring are retrieved in the T_1_ generation upon selection

For the collection of data required to build our in planta transformation compendium, a systematic review was conducted using Google Scholar and Scopus search engines to identify the bulk of research articles. Following the use of these tools, we complemented the compendium using articles initially found on ResearchGate and several other online web references such as EuropePMC. Due to the large number of research articles available for specific techniques (e.g. floral dip and pollen-tube pathway), we focused on identifying research articles that demonstrate the efficiency of these approaches in understudied plant species (i.e. all plants that are not considered commercial or model crops) to improve our global understanding of the applicability of these transformation strategies. On the whole, we manually curated, annotated, and reviewed 323 references (research articles, thesis, patents, etc.) tackling the topic of in planta transformation using this classification scheme (Table S1). In total, this compendium includes a total of 139 different species, 105 genera, and a broad range of techniques for each type of explant (Fig. 1; Table S1). All of the sections referring to specific in planta transformation techniques *de facto* refer to this compendium to limit the number of in-text references. For visualization, ggplot2 package version 3.3.5 with R version 4.0.4 [12] was used to build Fig. 1, whereas Figs. 2, 3, 4, 5, 6, 7, 8 and 9 were created with www.BioRender.com.

## Definition of in planta stable transformation

The act of generating stable plant transformants is a combination of two indissociable and interdependent steps: (i) the transformation of a plant cell; and (ii) the development of this cell into a whole plant [13]. In planta stable transformation, also called in situ transformation, techniques form a heterogeneous group of methods all aiming at performing the direct and stable integration of foreign T-DNA into a plant’s genome and regenerating the transformed cells into whole plants [5–11]. Unlike in planta transient transformation strategies, such as agroinfiltration, in planta stable transformation aims at generating heritable modifications using exogenous genetic material. In opposition to in planta strategies, in vitro indirect transformation/regeneration techniques, often called conventional transformation/regeneration methods, aim at regenerating an explant that produces a callus (i.e. a more or less developed unorganized plant structure made of parenchyma cells) under strictly sterile conditions [8].

Historically, the most common definitions of in planta transformation have been (i) a means of transformation without tissue culture step [5, 7] and (ii) a means of transformation of intact plants or plant tissues without callus culture or regeneration [14, 15]. In our opinion, these definitions are incomplete and not nuanced enough to take into account the broad diversity of available in planta methods. The challenging aspect of most in vitro indirect transformation/regeneration techniques stems from the combination of the hard-to-maintain micropropagation conditions and the callus regeneration step, more than the singular features of each aspect taken alone. As such, multiple highly efficient in planta research articles performing callus regeneration under in vivo conditions have been published over the years [16–20]. Similarly, several effective in planta protocols using minimal in vitro steps have also been published [21–25]. Conceptually speaking, these published methods all fall within the scope of in planta transformation and were self-described as in planta by their authors; however, their methods do not strictly follow the definitions mentioned above. Furthermore, several articles with major in vitro (e.g [26–28]) components have been published and were also self-described as in planta by their authors. These contrasting definitions underline the grey zone concerning the use of micropropagation within the realm of in planta transformation. For the purposes of this article, we redefined the in planta concept as the following: a means of plant genetic transformation with no or minimal tissue culture steps. To be considered minimal, the tissue culture steps should meet the following pivotal criteria: (i) short duration with a limited number of medium transfers; (ii) high technical simplicity (i.e. simple medium composition with a limited list of hormones); and (iii) regeneration using a differentiated explant that does not undergo a callus development stage and thus relies on direct regeneration.

## Classification of in planta transformation methods

In contrast to conventional transformation methods, in planta strategies are extremely heterogeneous in their modes of action and types of organ targeted. At present, there are hundreds of in planta protocols available in the literature. The classification of these protocols into a structured system is challenging due to numerous factors, including: (i) heterogeneous mode of action; (ii) skewed distribution of the publications between the methods (i.e. some methods have dozens of publications, while others have only one or a few); (iii) specific methods that have been reviewed thoroughly in the past while others are nearly absent from the literature; and (iv) the scientific pertinence/novelty versus the number of publications that are often uncorrelated.

This article has been written with the intent of finding a balance between all of these aspects, with an emphasis on techniques not thoroughly reviewed in the past. A large number of techniques presented in this paper were named/renamed by ourselves to distinguish them from similar techniques. As such, the names found in this paper might differ in other references. To build this review paper, we classified the references based on their explant of choice using the following nomenclature: (i) germline [female (ovule) and male (pollen) gametes]; (ii) embryo (aka zygotes); (iii) shoot apical meristem and adventitious meristems; (iv) vegetative tissues; and (v) novel systems (Table S2).

(i) *Germline transformation* techniques are regeneration-independent strategies that target the haploid female (egg) or male (sperm) gametophytic cells before their fusion and the subsequent generation of a diploid zygote [29, 30]. Germline-based transformation techniques can be divided into two categories based on the nature of the targeted sexual organ: (i) ovule (female organ) and (ii) pollen (male organ).
(ii) *Plant zygotes* are progenitor stem cells generated from the fusion of two haploid gametes, the egg and the sperm cells, from which all of the embryonic and post-embryonic organs are generated [31]. The zygote is divided into two parts, a small apical and a large basal cells [32]. Through the development of the embryo, the small apical part will give rise to the shoot meristem [32]. In this paper, the *embryo* section includes all the methods performed at the post-pollination stage until the emergence of the shoot apical meristem from the seed upon germination.
(iii) The *shoot and root meristems* are highly organized structures composed of proliferating embryonic-type cells involved in the continuous generation of aerial and underground plant organs through mitosis [33]. A portion of the stem cells present in these meristems are activated upon germination to produce primordia of lateral organs, while a pluripotent undifferentiated population is maintained at its center to ensure self-renewal and integrity [33]. Unlike the floral meristem, the stem cell features of the shoot apical meristem are maintained throughout the whole life cycle of the plant [34]. The protocols included in the pre-formed meristem sections include those that target different types of meristems (apical, axillary, or adventitious) upon their emergence from the seed until their senescence.
(iv) Callus refers to the accumulation of disorganized cell masses generally associated with the wounding of *vegetative tissues* [35]. These pluripotent cell masses either form roots or shoots through cellular reprogramming upon inductive cues (e.g. presence of light) [36]. Monocots and dicots have important biological differences that influence their respective abilities to form new meristems from a pluripotent callus mass [37, 38]. In dicots, most anatomical organs display the ability to generate calluses during the whole life of the plant, whereas monocots do not have a true vascular cambium with the ability to undergo cell rearrangement [37, 38]. Callus generation in monocots is limited to the base segment of leaves and the lateral and tip regions of roots [37]. As such, dicots are much more amenable to in vivo regeneration and propagation (e.g. grafting and cuttings) than monocots [37, 39].
(v) Two transformation techniques (i.e. grafting-mediated transformation and transformation using viral-based vectors) have been classified in the “*novel systems*” section because they harbor special features that limit their classification using the four other different types of explants. At present, the scope of these methods remains more limited than all of the other in planta strategies presented here due to specific experimental requirements.

## Means of in planta transformation

Plant transformation techniques can be divided into two main gene transfer categories: (i) direct gene transfer; and (ii) indirect gene transfer [40]. The former transfer strategy aims at introducing naked DNA into a plant genome through chemical or physical means (e.g. biolistics, electroporation, and polyethylene glycol), whereas the latter involves the introduction of DNA using biological vectors (e.g. *Agrobacterium spp*., *Ochrobactrum haywardense*, or viral vectors) [40]. *Agrobacterium tumefaciens*-mediated transformation is by far the most used method among the different in planta approaches as it is a simple and cost-effective option that generates few copy numbers in the generated transformants [11]. In addition, *Agrobacterium* is effective in a wide range of plant genotypes and species and can be used with various types of in planta strategies, thus making it a robust, reliable, and versatile transformation system [11]. *Agrobacterium rhizogenes* is generally used to perform in planta transformation that results in non-heritable changes through the formation of hairy roots in composite plants; however, the recently developed cut-dip-budding [41] and vine-cutting node inoculation [42] methods have demonstrated that *A. rhizogenes* can be used to perform stable transformation in asexually propagated plant species such as sweet potato (*Ipomoea batatas*). Although more marginal in their use, several other methods, such as direct DNA uptake [43] and biolistics [14, 44], are now sometimes used in diverse in planta protocols and are alternatives to *Agrobacterium*-based methods.

## Types of regeneration pathways

In most genetic transformation experiments, the regeneration of a positive somatic mutant cell into a whole plant is the rate-limiting step that is associated with the recalcitrant features of most hard-to-transform species [45]. In plants, this step can be undertaken using two strategies that are based on totipotency (i.e. a cell’s feature that enables it to dedifferentiate and redifferentiate into different tissues, organs, or whole organisms): (i) somatic embryogenesis; or (ii) de novo organogenesis [46]. Over the years, fertilization-based transformation techniques based on the transfer of exogenous DNA to male/female haploid gametes (e.g. floral dip) or fertilized diploid zygotes (e.g. pollen-tube pathway) have also been developed and are considered regeneration-independent [47]. In general, regeneration-independent techniques are often considered more efficient than their dependent counterparts due to their omission of the regeneration step; however, these approaches also have their own set of disadvantages including the generation of hemizygous (i.e. only one copy of a transgene at a given locus in an otherwise diploid cell) individuals when targeting haploid gametes [47].

### Somatic embryogenesis

Somatic embryogenesis is a mechanism in which differentiated cells undergo dedifferentiation to become embryonic stem cells [48, 49]. Following this step, embryonic stem cells can differentiate into meristematic cells to become a single and viable plant [48]. In the literature, the main difference between somatic embryogenesis and indirect de novo organogenesis is the presence of a somatic embryo formation step in the former, whereas the latter undergoes a callus generation step [48]. As such, both mechanisms require a regeneration step to form a new plant. To our knowledge, somatic embryogenesis, either through the direct or indirect pathways, is not a mechanism used for in planta transformation due to its extensive tissue culture requirements. In consequence, the term regeneration-dependent strategies will refer herein to only methods using a de novo organogenesis mechanism.

### *De novo* organogenesis

Plant regeneration occurs upon cell wounding and aims at repairing or replacing the damaged anatomical structures using totipotency and pluripotency, which will lead to the subsequent generation of adventitious organs [48]. Adventitious organs are defined as either root or shoot meristematic buds that arise from growing areas that typically do not contain such organs [50]. In the literature, no specific terms distinguish the adventitious organs which are obtained either from indirect or direct de novo organogenesis [48]. In addition, indirect and direct shoot regeneration events often occur simultaneously upon wounding [16], a phenomenon that can generate some confusion between the mechanisms in the literature. However, the distinction between both types of adventitious shoot formation pathways is important due to major differences in their underlying biological mechanisms and impacts on the transformation event. For instance, direct regeneration strategies, both under in vivo and in vitro conditions, can instigate a varying degree of chimerism in the transformants, thereby creating heterogenomic mutants that will require subsequent segregation to recover non-chimeric plants [51, 52]. In plants obtained with indirect organogenesis, chimerism is less concerning because single-cell regeneration can be undergone using a selection marker (e.g. antibiotics or herbicides), but somaclonal variations are typically more prevalent [53].

Overall, techniques using a de novo organogenesis approach can be classified based on their use of tissue culture (i.e. in vivo/tissue culture-independent vs. in vitro/tissue culture-dependent) and methods of regeneration (i.e. direct regeneration vs. indirect regeneration) [54] (Fig. 2). In general, in planta strategies aim at limiting tissue culture to a minimum and consequently either use in vivo direct regeneration or in vivo indirect regeneration strategies. From a technical standpoint, the in vitro direct regeneration pathway can be considered a crossover between the in vitro direct regeneration and in vivo indirect regeneration concepts as the explants are micropropagated under sterile conditions but regenerated through direct organogenesis. Although not considered *in planta per se*, the protocols using the in vitro direct regeneration pathway generally have a faster regeneration rate (often between 4 and 8 weeks), lessened use of hormones, higher success rates, greater genotype-independency, and decreased technical skills requirements [55, 56]. A short section of this paper will be dedicated to the methods using this pathway since those offer a promising alternative to the in vitro indirect regeneration pathway, particularly in monocots [57, 58].

## Germline transformation

### Floral dip and similar methods (Ovule)

The most important contributor to the spread of the in planta conceptual framework is undoubtedly the floral dip method in *Arabidopsis* [59]. At its essence, the floral dip method is a simple and reliable method that aims at performing germline transformation through the dipping of developing floral tissues into resuspended *Agrobacterium* inoculum [59] (Fig. 3a). The first iteration of this method was developed by Bechtold and Pelletier [60] using vacuum-infiltration of the floral organs. Despite its high transformation rates, this protocol was largely supplanted by the protocol proposed by Clough and Bent [59] which removed the vacuum-infiltration step and replaced it with a simple dip into a solution containing *Agrobacterium*, sucrose, and a surfactant (i.e. Silwet L-77), thus streamlining the technical aspect of the method and increasing the speed of the procedure. As such, the approach developed by Clough and Bent [59] is now the mainstay for transforming *Arabidopsis*, a popularity largely due to its high transformation efficiency as rates between 0.1 and 3% are typical^1^ [61]. Over the years, other iterations of the technique, such as the floral dip with low inoculum density [62], vacuum-infiltration of closed floral buds [63], and simplified floral dip [64], have been proposed to upgrade specific aspects of the method. Although the floral dip approach is a common technique for plant transformation, two factors still readily limit its development on a broader scale: (i) the generation of hemizygous offspring; and (ii) a narrow range of species amenable to the method.

Hemizygous offspring are generated with the floral dip method since the transformation event happens after the divergence of anther and ovary cell lineages in *Arabidopsis* [47]. In *Arabidopsis*, the stigmatic cap forms over the top of the gynoecium, enclosing the locules 3 days before anthesis [47]. As a consequence, the primary targets of the floral dip method are the female reproductive organs, the ovules, and embryo sacs, whereas the pollen or pollen tubes remain untouched [47]. To segregate all hemizygous progenies and recover only offspring with homozygous genotypes, a thorough screening must be performed until the T_3_ generation as the progenies from the T_2_ generation are not stable [65, 66].

Although tremendous research has been pursued on the floral dip method, the number of species amenable to this technique remains modest in comparison to other techniques, such as the shoot apical meristem injury approach. At present, the bulk of the floral dip protocols have been developed for species belonging to the *Brassicaceae* family, but transformation procedures based on this approach have also been demonstrated to be efficient for 12 other families (e.g. Linaceae and Solanaceae) (Table S1). Still, the protocols targeting species belonging to families other than *Brassicaceae* are sparse and generally less efficient due to lower transformation rates, cumbersome manipulations, and complicated technical requirements (e.g. tomato/*Solanum lycopersicum* [67]). Numerous biological and morphological factors have been suggested to explain the limited expansion of the floral dip technique to other plant species, including physical barriers associated with flower morphology [61], necrotic reaction to the presence of *Agrobacterium* causing abortions in the flowers [61], lower seed set [68], reduced susceptibility to *Agrobacterium* [68], and bigger size of the plant and/or flower structures [5]. Over the years, modifications to the floral dip method have been developed to increase its efficiency with plant species that are not members of the Brassicaceae, while retaining the core concepts of the strategy. Amongst these innovative strategies are the floral bud injection (tomato, poplar/*Populus sp.*, chickpea/*Cicer arietinum* and sunflower/*Helianthus annuus*) [69–72], floral bud painting (maize/*Zea mays* and tomato) [67, 73], and floral bud spray (*Arabidopsis*, wheat/*Triticum aestivum*, and Indian mustard/*Brassica juncea*) strategies [74–76].

### Pollen transformation

In the pollen transformation method, the desired foreign gene is introduced into the pollen grains via *Agrobacterium* or directly with naked DNA [77] (Fig. 3b). Following this step, the transformed pollen grains are subsequently used to pollinate the stigma and fertilize the recipient egg *in vivo.* Pollen grains are an interesting target for transformation as they can be easily isolated, occur in large numbers, and can be easily transformed [77]. Pollen grains harbor a coat derived from the anther tapetum (the pollenkitt/tryphine), an outer thick cell wall (the exine), and a thin inner cell wall (the intine), that block the integration of exogenous DNA [77]. In addition, germinating pollen grains release nucleases that catalyze the cleavage of phosphodiester bonds between nucleotides of nucleic acids [78]. In combination, the thick wall/coat and release of nucleases limit the use of conventional transformation methods to integrate the transgene into the pollen grain [77, 78]. To circumvent this problem, various methods such as electroporation [79], particle bombardment [80, 81], vacuum infiltration [82], sonication [83], *Agrobacterium* [82, 84], and magnetofection [85, 86] have been used to facilitate the introduction of transgenes into pollen grains or microspores, with varying degrees of success. Several transformation methods based on pollen incorporate a short in vitro period at the beginning of the experiment as in the case of the male germline transformation (MAGELITR) system [81], which can be a limiting factor for labs without access to micropropagation facilities. Overall, pollen transformation has been demonstrated to be efficient in several species, including tobacco [79–81, 87], cotton (*Gossypium hirsutum*) [82], sorghum (*Sorghum bicolor*) [88], petunia (*Petunia x hybrida*) [89], Indian mustard [83], and maize [90], but its implementation remains challenging in a large number of species, with contrasting results between different labs (e.g. magnetofection was reported to be inefficient in monocots [91]).

## Embryo

### Pollen-tube pathway

The pollen-tube pathway strategy aims at applying exogenous donor DNA onto the severed style of the recipient plant, which will be transported via the growth of the pollen tube to the ovary [92] (Fig. 4a). Reaching the ovary, the foreign DNA will be integrated into the undivided recipient zygote, thus leading to the generation of a transformed embryo [92]. To improve the rates of transformation, researchers often cut the styles of the recipient plant [92]. The pollen-tube pathway transfer technique is one of the oldest transformation techniques that has been investigated, with reports dating back to 1983 in cotton [93] and 1989 in rice (*Oryza sativa*) [94]. Although beneficial in many aspects (e.g. no regeneration step and fast preparation), this method has also demonstrated some limitations in the past, such as poor transformation efficiency [95, 96] and a lack of reproducibility [97–99], which led to a rise in skepticism regarding some of its claimed benefits (e.g. universal application) [92]. For instance, Li et al. [99] have observed many inconsistencies with soybean (*Glycine max*) plants treated with the pollen-tube pathway technique. In their experiments, all the plants exhibiting positive β-glucuronidase (GUS) activity were found to be untransformed when analyzed using polymerase chain reaction (PCR). Similarly, morphological variation was observed in the first generation of some plants, but not in the subsequent generations. As a consequence of these inconsistent results, there has been a disinterest in this transformation system in the Western hemisphere [92]. In the meantime, China continued to improve the procedure and has now developed broad expertise with this transformation strategy, resulting in a significant proportion of the research articles only being available in Mandarin [100]. When compiling the research articles for this review, we found that a broad selection of protocols is now available for this strategy with dozens of research articles published for major commercial crops, including cotton [101–103], maize [104], rice [105], and wheat [106], as well as for at least 24 other species.

### Ovary-drip

The ovary-drip method differs from the pollen-tube pathway as the exogenous DNA (i.e. which is supplied under the form of a minimal linear gene cassette) is directly delivered to the ovule after pollination with the complete removal of the style [107] (Fig. 4b). Generally, the ovary-drip method has higher transformation rates than the pollen-tube pathway (e.g. 3.38% transformation frequency with the ovary-drip method vs. 0.86% with the pollen-tube pathway [108]), but requires careful manipulation to limit the risk of mechanical damage to the ovule [92, 109]. This method has been used successfully to transform soybean [107, 110] and maize [111, 114]. One of the key factors influencing the success rate of this method is the length of the style. Liu et al. [112] investigated the optimal length of the soybean style and found that the complete removal of the style without ovary wounding generated the highest proportion of transformants, 11%.

### Pollen-tube agroinjection

At its core, the pollen-tube agroinjection method combines the principles of the pollen-tube injection pathway with *A. tumefaciens*-mediated transformation (Fig. 4c) [113]. In this method, carinas (i.e. two conjoined lower petals of a legume flower that enclose the stamen and style) of freshly opened flowers (in this case peanut) need to be punctured using injector needles and injected with 0.1 mL of resuspended *Agrobacterium* solution. The method was used to generate transgenic peanut lines encoding the peanut *BAX INHIBITOR-1* gene with an overall transformation rate of 50%. To the best of our knowledge, only one research article using this approach has been published, but the high transformation rates suggest that it might be an efficient alternative to the conventional pollen-tube pathway technique.

### Ovary injection transformation

The ovary injection method aims at injecting *Agrobacterium* directly into the locule of a plant’s ovary to reach the embryo using a micro-injector or a syringe after pollination (e.g. cotton [114]) (Fig. 4d). This method has been used with success in about ten species, but has been demonstrated to be particularly effective in tomato [118–117] and, to a minor extent, soybean [118]. In tomato, Hasan et al. [116] developed a protocol in which mature and ripe fruits were injected with 1 mL of an *Agrobacterium* solution containing a GUS reporter and incubated at 28 °C for 48, 72, and 96 h. The highest number of stable transformed plants was obtained with a 48 h incubation period, with 88% being positive for the GUS assay. Using a similar protocol, Yasmeen et al. [115] obtained transformation rates of 35–42% in tomato depending on the construct. When injecting the *Agrobacterium* solution at stage I (i.e. 2–3 days after pod formation) in soybean, transformation efficiencies between 6.45 and 14.2% and 28.75–35.48% were respectively obtained using GUS assays on plants and seeds [118]. To improve the transformation rates of the ovary injection method, a similar method using micro-vibration was developed by Liou [119]. In this approach, the stigma of the flower is removed and exogenous DNA is injected through the cut-off position and toward the locule inside the ovary. Following this step, a micro-vibration treatment will be performed with an ultra-sonic device to favor the placement of DNA around the ovule and improve integration.

### Infection of pre-imbibed embryos with *agrobacterium*

The infection of pre-imbibed embryos with *Agrobacterium* is a simple technique in which a seed is injured (e.g. seed pricking, tip cutting, sonication, or puncturation) and then imbibed to facilitate the infection of the embryo by *Agrobacterium* (Fig. 5a). This technique was first developed by Graves and Goldman [121] by pricking four-day-old germinating maize seeds four times in an area extending from the scutellar node through the mesocotyl to infect the cells located in this zone with *Agrobacterium*. Subsequently, a method for the transformation of soybean was developed using a similar approach [121]. In the Chee, Fober, and Slightom [121] protocol, imbibed soybean seeds with one cotyledon removed were pricked at three different points into the plumule, cotyledonary node, and adjacent regions and injected with 30 µL of *Agrobacterium* culture at each injured point. The observed transformation rates obtained with this method were 0.7% in the R_0_ plant and 0.07% in the R_1_ generation. Although the rates of transformation were low for both of these protocols, they paved the way to more performing protocols in a large number of species. Following the development of the Graves and Goldman [120] method in maize, a variant involving the use of uninjured seeds was developed in 1987 using *Arabidopsis* [122]. In this protocol developed by Feldmann and David Marks [122], *Arabidopsis* seeds were imbibed for 6, 12, or 24 h following a one-step or two-step imbibition protocol, infected with 3 mL of an overnight culture of *Agrobacterium* and co-cultivated during 24 h before being washed with sterile water. Subsequently, the seeds were sown on vermiculite pre-soaked with a complete nutrient solution. Although the transformation efficiencies were rather low (0.0015-0.3200%), the protocol still demonstrated that it was possible to generate transformants without causing any injuries to the pre-imbibed seeds.

### Agro-imbibition

The agro-imbibition technique is a relatively new approach that aims at fully imbibing whole seeds with an *Agrobacterium* solution to infect them (Fig. 5b). The method is simple and has a reduced workload; however, seven patents have been deposited for this method, suggesting that a license might be required to use it [123]. In their recent article, Kharb et al. [123] detailed the core principles of this genotype-independent in planta strategy. In their protocol, seeds are surface sterilized using a 0.1% HgCl2 solution for 10 min, imbibed in a resuspended culture of *Agrobacterium* (O.D. = 0.6) with shaking at 100 revolutions per minute (RPM), and then germinated on a simple germination medium containing 250 mg/L cefotaxime or on soil. According to the authors, many species (e.g. chickpea, pigeon pea/*Cajanus cajan*, wheat, soybean, and rice) are amenable to this approach, with efficiencies ranging from 14.3% in chickpea up to 93.8% in rice.

### Imbibition of desiccated embryos

This approach aims at rehydrating desiccated zygotic embryos with an *Agrobacterium* solution [125] (Fig. 5c). Upon desiccation, several physiological modifications (e.g. bursting of the cell walls) occur which facilitate the integration of DNA in the zygotic embryo [126]. Consequently, dry cells become permeable to large plasmid DNA molecules and transformation can happen without relying on *Agrobacterium* [126]. In addition, cellular permeabilization agents (e.g. toluenes) can be used to improve the proportion of DNA intake [127]. Arias et al. [125] developed a protocol in which soybean embryonic axes (i.e. zygotic embryos) were imbibed in an aqueous solution for 18 h and subsequently desiccated at room temperature until reaching a moisture content of 10–25%. After desiccation, the zygotic embryos were imbibed again with an *Agrobacterium* solution for approximately 2 h at room temperature. Arias et al. [125] indicated transformation rates between 0 and 80% in T_0_ mutants using GUS assays and mentioned that T_3_ transformants were generated for the pBPSLM003 and pCAMBIA3301 plasmids with this method, thus indicating that the method can be efficiently used to generate stable transformants. In addition, the method has also been proven to be compatible with *Arabidopsis* [125].

## Shoot apical and adventitious meristems

### Shoot apical meristem injury under in vivo conditions

The shoot apical meristem is one of the primary targets of in planta transformation, and an extensive literature targeting this organ under in vivo growing conditions is available. All plant species display at least one form of shoot apical meristem [128], and the transformation of this organ can be performed at almost any stage of a plant’s life, from the seedling to the adult stages [8]. Together, these two characteristics (i.e. all stages of growth and all plant species) contribute to the universal applicability of the shoot apical meristem injury transformation approach [8, 128]. On the whole, the strategies grouped under this approach loosely share four core concepts that are: (i) wounding the apical meristem region using a needle, scalpel, syringe, or another method (e.g. sonication); (ii) infecting the meristem with *Agrobacterium*; (iii) growth of the seedlings under in vivo conditions for most of their lifecycle; and (iv) chimeric T_0_ generation with selection in the T_0_ (rare) or T_1_ (standard) generation [129] (Fig. 6a). A standardized protocol named apical meristem targeted in planta transformation, which was first validated in safflower (*Carthamus tinctorius*) and peanut respectively by Rohini and Sankara Rao [130] and Rohini and Sakanra Rao [131], was proposed as a low-tech efficient transformation method that can be applied to both dicots and monocots. In this standardized method, the differentiating apical meristem region of two-day-old seedlings is injured using a needle and subsequently infected using an *Agrobacterium* solution supplemented with Winans’ AB minimal and wounded tobacco leaf extract [129, 132]. After the infection, the plants are transferred to autoclaved soilrite and allowed to grow for ≈ 1 week under a 16 h photoperiod [129]. Following this step, the plant is transferred to pots and allowed to set seed. The T_1_ offspring of these chimeric plants are subsequently screened using a selectable marker such as antibiotic resistance and/or PCR amplification [129]. Overall, the transformation efficiencies can be quite high considering the simplicity of the approach. For example, the transformation efficiencies were respectively evaluated to be 5.3% and 1.3% in the cultivars ‘A-1’ and ‘A-300’ using histochemical assays, PCR amplification, and Southern blot analyses in T_0_ and T_1_ safflower plants [130]. In peanut, the transformation frequencies were evaluated to be 3.3% based on histochemical assay and by PCR analysis of the *GUS* gene [131].

Over the years, several variations have been incorporated into this standard protocol to improve the rate of transformation. For example, the generation of mosaic plants in the T_0_ generation requires a stringent screening of the transformants to be performed in the T_1_ generation. In some protocols, a selection step under in vivo conditions (e.g. maize [133]) or in vitro conditions using soilrite as a medium (e.g. roselle/*Hibiscus sabdariffa* [134]) has been added after inoculation to select the best-performing T_0_ chimeric plants. The addition of this selection step limits the number of plants that will be cultivated until the T_1_ generation and improves the overall rate of transformation. Similarly, some protocols have incorporated steps to improve the injury step by adding sonication (e.g. horse gram/*Macrotyloma uniflorum* [25]), electroporation (e.g. pea/*Pisum sativum*, soybean, cowpea/*Vigna unguiculata*, and lentil/*Lens culinaris* [135, 136]), and/or vacuum infiltration (e.g. *Arabidopsis* [137], barrel clover/*Medicago truncatula* [138], cumin/*Cuminum cyminum* [139], mung bean/*Vigna radiata* [140] and horse gram [25]) procedures. Additional modifications include: (i) optimization of the *Agrobacterium* inoculum optical density (e.g. pigeon pea [141]); (ii) optimization of the acetosyringone concentration (e.g. tuberose/*Polianthes tuberosa* [142]); (iii) addition of a pre-culture step on Murashige and Skoog (MS) medium before inoculation (e.g. chickpea [143]); (iv) addition of a co-cultivation step on MS medium after inoculation (e.g. radish/*Raphanus sativus* [144]); and (v) use of a germination medium under in vitro conditions (e.g. sesame/*Sesamum indicum* [145]).

### Plumular meristem strategy

Amongst the different protocols using direct de novo shoot organogenesis, the plumular meristem strategy was proposed as a time-efficient direct regeneration-based transformation approach with high transformation rates for chickpea [22, 146] and pigeon pea [147, 148]. In this system, three-day-old seedlings are decapitated at the shoot apex and pricked in the apical portion and cotyledonary nodes [22, 147] (Fig. 6b). After co-cultivation with *A. tumefaciens*, multiple shoot induction is performed through the transfer of the explants on a sterile MS medium containing 6-benzyl amino purine (BAP) and 1-naphthaleneacetic acid (NAA) for three days. Following this step, the plants are moved to pots and grown under greenhouse conditions until reaching the T_1_ generation. The transformation rates using the plumular meristem strategy method were 44% and 72% in the T_1_ generation of chickpea [22] and pigeon pea [147], respectively. A similar protocol to the plumular meristem method was developed for alfalfa (*Medicago sativa*) [149]. In this protocol, three-day-old alfalfa seedlings are excised at the cotyledonary attachment region of the hypocotyl and wounded by vortexing with sterile sand. Following the excisions, the plants are transferred to a hormone-free medium for a short recovery time and cultivated in vitro for 14 days in a half-strength MS medium containing timentin. After this cultivation step, plants are transferred to greenhouse conditions for further growth. When performing this protocol, Weeks et al. [149] observed that excisions performed below the unifoliate leaf base eliminated the potential for shoot recovery, whereas those performed at or above the apical node resulted in the growth of new shoots in 95% of the cases. Using this protocol, about 7% of the seedlings produced progenies segregating for the T-DNA [149].

### Propagule transformation

Several specialized vegetative plant organs involved in asexual reproduction, often called vegetative propagules, are ideal targets for in planta transformation due to the rapid development of growing permanent plant tissues from actively dividing meristematic cells through mitosis [150]. Propagules include stem tubers (e.g. potato and yams), tuberous roots (e.g. sweet potato and dahlia), root suckers (e.g. apple, pear, blackberries, and raspberries), runners (e.g. strawberries), bulbs (e.g. onions, tulips, and lilies), and plantlets (e.g. mother of thousands/*Kalanchoe daigremontianum*) [151]. The cut–dip–budding delivery approach aims at actively regenerating shoots from adventitious buds developed from root suckers transformed with *Agrobacterium rhizogenes* under in vivo conditions [41] (Fig. 7a). This strategy has been demonstrated to be efficient with ten cultivars of sweet potato, two herbaceous plants (i.e. rubber dandelion/*Taraxacum kok-saghyz* and crown vetch/*Coronilla varia*), and three woody plants (i.e. Chinese sumac/*Ailanthus altissima*, Japanese angelica tree/*Aralia elata*, and glorybower/*Clerodendrum chinense*). Using this approach, the observed transformation efficiencies were 10–47% for sweet potato, 40–50% for *T. kok-saghyz*, 3% for *C. varia*, 39% for *A. altissima*, 2% for *A. elata*, and 48% for *C. chinense* [41]. A similar in vitro protocol based on the regeneration of shoots from *A. rhizogenes*-infected hairy roots has been demonstrated to be efficient in apple (*Malus pumila*) and kiwi (*Actinidia chinensis*), with a short regeneration time of about 9–11 weeks [152]. The Regenerative activity-dependent in planta injection delivery (RAPID) method aims at generating transformants from infected renascent tissues of sweet potato, potato (*Solanum tuberosum*), and bayhops (*Ipomoea pes-caprae*) under in vivo conditions [150] (Fig. 7b). In this protocol, stable transformation is obtained through the delivery of *A. tumefaciens* to the stem by injection and subsequent vegetative propagation of the emerging positive tissues from the wound site. Selection of the positive tissues is performed through molecular detection and/or phenotypic analysis if using a visual selection marker. Overall, the RAPID protocol displayed a short transformation time, between three to ten weeks, with a high transformant acquisition rate of 28–40%. Additional systems for propagule transformation have been developed for banana (*Musa. sp.)* suckers [153], gemmae of umbrella liverwort (*Marchantia polymorphya*) [154], leaf notches of cathedral bells (*Kalanchoe pinnata*) [155], sugarcane (*Saccharum spp.*) setts [156], and bulbs of the *Notocactus scopa* and *Hylocereus trigonus* cacti [157].

### Exogenous morphogenic regulators and direct delivery

In recent years, the use of exogenous morphogenic regulators has also been explored as an efficient option to induce de novo shoot organogenesis. Morphogenic regulators, such as *LEAFY COTYLEDON 1* [158, 159], *LEAFY COTYLEDON 2* [160], *BABY BOOM* [161] *and WUSCHEL* [162], are key genes involved in a plethora of functions such as plant morphogenesis and regeneration [163], de novo establishment of shoot stem cell niche [164], shoot and root meristem homeostasis [165] and shoot apical establishment [166]. As such, their expression is critical for de novo shoot organogenesis. Morphogenic regulators promote the production of somatic embryos or embryo-like structures on vegetative or callus explants, an effect that is increased upon overexpression [167–169]. Current reports have demonstrated that ectopicly expressed morphogenic regulators can be harnessed to improve the in vitro recovery rates of transgenic calli from hard-to-transform genotypes of at least 12 commercially important monocot species (e.g. rice) [170–174]. Despite the observed increase in the regeneration rates of transgenic calli [170], the in vitro use of ectopically expressed morphogenic regulators still remains challenging on a technical level.

To overcome these limitations, Maher et al. [175] and Cody et al. [176] developed an exogenous morphogenic regulator-based in vivo transformation method called Direct Delivery. In opposition to the Fast-treated *Agrobacterium* co-culture (Fast-TrACC) method (i.e. a similar method with an in vitro phase), the Direct Delivery entirely sidesteps tissue culture [176]. In the Direct Delivery method, developmental regulators, such as maize *WUSCHEL/WUSCHEL 2* (*Wus2*), cytokinin *ISOPENTYL TRANSFERASE* (*ipt*), and *A. thaliana SHOOT MERISTEMLESS* (*STM*), and gene-editing reagents are directly delivered with *Agrobacterium* to somatic cells of whole plants to induce the formation of de novo meristems [175, 176] (Fig. 7c). Following the injection of *Agrobacterium*, visible meristems are removed and shoot formation occurs at the wound sites after 38–48 days [175, 176]. Maher et al. [175] demonstrated that this approach generates high transformation rates with tobacco/*Nicotiana benthamiana* (i.e. gene editing efficiencies ranging from 30 to 95%) and observed positive results with potato and grapevine (*Vitis vinifera*) under in vitro conditions. Lian et al. [177] successfully regenerated snapdragon (*Antirrhinum majus*) and tomato shoots using a protocol similar to Direct Delivery under in vivo conditions but with the *PLETHORA (PLT5)* developmental regulator. With this ectopic expression approach, transformation efficiencies up to 11.25% and 13.3% were obtained for snapdragon and tomato, respectively [177]. The same test was performed on cabbage (*Brassica rapa*) and sweet pepper (*Capsicum spp.*) in vivo, but possibly failed due to the rapid deposition of suberin and lignin in response to wounding [177]. Direct delivery was also performed on apple (*Malus pumila*) and grapevine by Spicer [178], but without observing gene edits in the generated shoots.

### Nodal agroinjection

The nodal agroinjection approach is a simple method that aims at injecting resuspended *Agrobacterium* in the first and second nodes of cotyledonary branches. This strategy was first validated by Wang et al. [179] in peanut and subsequently used by Han et al. [180] to generate CRISPR-Cas9 knockout peanut mutants for the *FATTY ACID DESATURASE 2B* (*AhFAD2B*) gene. In the original protocol, 5 µL of *Agrobacterium* was injected into the nodal sections of 30-day-old peanut plants. From the 820 plants recovered with this method, a total of 371 (45.24%) were PCR-positive.

### Direct regeneration of embryos and shoot apical meristems under in vitro conditions

This direct regeneration strategy aims at regenerating the meristematic cells of a differentiated explant under in vitro conditions (Fig. 8a). Both embryonic axes and developed shoot apical meristems have been demonstrated to be suitable explants for direct organogenesis under in vitro conditions. The use of a differentiated explant typically hastens the shoot regeneration rate, diminishes the requirements in hormones, simplifies medium composition (i.e. often only sucrose), and increases the resilience of the explant toward *Agrobacterium* overgrowth [55, 56, 181]. A large literature search has demonstrated the efficiency of several transformation/regeneration systems for the embryonic axes of watermelon [182], field bean [183], cowpea [184], chickpea [185, 186], common bean [184, 187], black gram (*Vigna mungo*) [188, 189], purslane [190], eggplant [191], and snake gourd (*Tricosanthes cucumerina*) [27]. Two of the most commonly transformed species using the in vitro embryonic axis method are soybean and cotton, sometimes with innovative technical aspects. For example, Paes de Melo et al. [55] and Ribeiro et al. [56] have respectively proposed protocols in which soybean and cotton embryonic axes are injured using biolistics and subsequently infected with *Agrobacterium*. In their protocols, shooting, rooting, and selection are subsequently performed simultaneously in a medium containing 6-benzylaminopurine (BAP) and activated charcoal. In this system, transformants are selected with the selection marker gene *AHAS* which confers resistance to the systemic herbicide Imazapyr. Using these protocols, Paes de Melo et al. [55] and Ribeiro et al. [56] have obtained transformation efficiencies averaging 9.84% for soybean and 60% for cotton. Similarly, several shoot apical meristem-based transformation/regeneration systems have been demonstrated in many dicots (e.g. cucumber [192], petunia [193], camelina/*Camelina sativa* [194], Dalmatian chrysanthemum/*Tanacetum cinerariifolium* [195] and cotton [196, 197]) and monocots (e.g. wheat [14, 44, 198], finger millet/*Eleusine coracana* [199], foxtail millet/*Setaria italica* [200], pearl millet/*Pennisetum glaucum* [201] and rice [57, 58, 202, 203]). In addition, an extensive literature dedicated to the in vitro regeneration of embryonic axes or excised shoot apical meristem without transformation is available for a large number of species (e.g. finger millet [204, 205], maize [206] and rice [207]). These regeneration protocols serve as a basis for the development of new transformation methods as those could be converted with minimal effort. Overall, these in vitro systems offer numerous benefits over many of the in planta systems and are one of the most interesting alternatives to streamline transformation in monocots. However, these methods require access to micropropagation facilities and are technically more challenging than most in planta techniques.

## Vegetative tissues

### Callus-based transformation system

In transformation systems using an in vivo callus-based approach, seedlings or mature plants are injured and their wounds are treated using a solution of *Agrobacterium* [16, 54] (Fig. 8b). Following this step, the injuries are subjected to hormone treatment, if necessary, to promote the development of a callus and/or adventitious buds [16]. In some cases, selection by treating the wounded area using a selection marker (i.e. antibiotic or herbicide) is performed to identify the putative transformants [16]. In transformed tomatoes, Pozueta-Romero et al. [208] observed that proper kanamycin selection favors the competition of transformed over untransformed cells during de novo shoot organogenesis, thus increasing significantly the number of regenerated transformed shoots. To promote callus growth, inoculated wounds can be covered with parafilm, aluminum foil, mud, or plastic to maintain proper humidity, and adequate temperature and to provide a dark treatment, as darkness has been demonstrated to favor the development of callus mass [16, 17]. Over the years, in vivo callus transformation and/or regeneration has been demonstrated to be feasible in a broad range of fruit trees (e.g. orange/*Citrus sinensis* [20, 209], longan [19, 210], and pomelo/*Citrus maxima* [17, 209]), vines [passionfruit [16]], shrubs/trees (e.g. poplar [211–213] and eucalyptus/*Eucalyptus sp.* [211]) and perennial dicots cultivated as annual (e.g. tomato [18, 208]). In their patent, Mily et al. [18] also mentioned that soybean and coffee (*Coffea sp.*) generate new shoots upon decapitation and that chili pepper, eggplant (*Solanum melongena*), and common bean also display excellent regeneration and *GUS* expression abilities. Often, plants regenerated using this system will concomitantly undergo direct regeneration events (e.g. tomato and several relatives [214–217], soybean [218], and peanut [51]) which can lead to some form of mosaicism in the transformed plant (Fig. 8c). Although the literature for this technique is relatively sparse in comparison to other transformation strategies, a plethora of protocols using indirect de novo shoot induction without transformation are currently available for species such as poinsettia (*Euphorbia pulcherrima*) [219], tomato [216, 220–222], and chili pepper [208]. In addition, indirect de novo shoot induction without transformation has been validated in lignified woody jujube [54, 223–225] and pomegranate (*Punica granatum*) [226] trees under field conditions for colchicine mutagenesis treatments, thus demonstrating its versatility and potential.

## Novel systems

### Grafting-mediated transformation

At present, only one technique, named grafting-mediated genome editing, has been developed as a systematic in planta transformation tool to induce precise modifications in the genome [227] (Fig. 9a). In grafted plants, the formation of a successful graft union requires several steps, including the (i) lining of the vascular cambium; (ii) wound healing; (iii) formation of a callus bridge between the rootstock and the scion; (iv) generation of vascular cambium; and (v) development of the secondary xylem and phloem [228]. The formation of a callus bridge enables the horizontal gene transfer of phloem-mobile protein-coding RNAs through the phloem vasculature of grafted plants [229]. In 2016, Zhang et al. [230] demonstrated that transcripts harboring distinctive tRNA-like structures can move from a transgenic rootstock to a wild-type scion and be translated into proteins after transport. Taking advantage of this discovery, Yang et al. [227] investigated the generation of stable gene-edited plant lines using intraspecific and interspecific grafting in wild-type *Arabidopsis* and *Brassica rapa* to generate heritable modifications. To do so, phloem-mobile tRNA-like sequences were fused to Cas9 and guide RNA (gRNA) sequences to induce transport from the provider transgenic rootstock to the recipient scion through root-to-shoot movement. Using this system, the inheritance of deletion edits was 1.6% for heterozygotic and 0.1% for homozygotic genotypes, although the authors underline that these numbers were probably underestimated because the seedlings were screened in pools using PCR. As the T_0_ generation is chimeric, segregation must be performed in the subsequent generation to recover non-chimeric lines. To circumvent the step involving the generation of the mutant rootstock in recalcitrant species, the authors suggest using *A. thaliana* and *Nicotiana sp.* as rootstocks due to their simple and reliable transformation protocols and their very wide range of compatible distantly related species, including soybean and fava bean [39].

### Viral-based vectors

Virus-induced gene silencing (VIGS) is a method that uses modified viral vectors to induce transient gene silencing in plants [231, 232]. This technique allows for efficient gene function analysis but is generally not considered as a reliable method to generate stable mutations in plants although some shreds of evidence suggest that the silencing effect can be transmitted to the next generation [233]. To circumvent this issue, the virus-induced genome editing (VIGE) method was developed as a means to generate permanent mutations for the production of true-breeding lines [234]. The scope of action of viral-based vectors significantly increased with the development of genome editing technologies as the expression of short RNA sequences (e.g. gRNA) can be readily performed with the use of in planta Agrobacterium transient transformation strategies (e.g. agroinfiltration, agroinjection, agrospray, agrodrench, and rub inoculation) [235, 236]; however, heritable mutations are challenging to generate due to the seclusion of viruses from the meristematic cells of the shoot apical meristem but have been reported on rare occasions (e.g. Tobacco rattle virus [234] and Barley stripe mosaic virus [237]). To obtain a greater efficiency at generating heritable genome editing events, Ellison et al. [238] fused gRNA sequences to mobile *FLOWERING LOCUS T* (*FT*) sequences and cloned them into a Tobacco rattle virus vector (Fig. 9b). The resulting vector was subsequently inserted into the cells of Cas9-overexpressing tobacco plants via agroinfiltration. In its natural state, endogenous *FT* sequences move to the shoot apical meristem to induce flowering via the phloem upon transcription in the leaf tissues [239]. This characteristic enables the gRNAs to enter the shoot apical meristem upon the transcription of *FT*, thus generating stable mutations in the future offspring without relying on tissue culture. Following the publication of Ellison et al. [238], this versatile editing system has been confirmed to be also compatible with the Barley yellow striate mosaic virus [240] and Cotton leaf crumple virus [241, 242].

## Conclusion

Since the first reports of in planta transformation in the 1980s [122], hundreds of in planta protocols have been developed for a large number of species. The classification of these protocols into a structured system is challenging due to the broad range of approaches. However, much of the strength of the in planta concept lies in this heterogeneity and high diversity since it aims to work with the natural biological and morphological features of each species instead of trying to “force” the transformation process through challenging regeneration steps. The high level of versatility, decreased upfront cost, and reduced technical requirements of many of these techniques demonstrate the importance of this field of research for the progress of plant science. Still, many of these techniques require more extended research to validate their use in a broad range of species. For instance, de novo shoot induction using tissue culture-independent approaches seems to be a promising strategy for dicot transformation, particularly for species with a long juvenile period (e.g. fruit trees). The methods are simple, cost and time-efficient, mostly genotype-independent, reliable, and based on prior knowledge from tissue culture-based de novo shoot induction methods. Furthermore, the protocols can be adapted for a wide range of experimental settings (e.g. lab vs. field conditions) and plant developmental stages (e.g. younger seedlings vs. lignified woody plants). Theoretically, this approach boasts all the most important features for a transformation method; however, it seems largely unexplored in the literature in comparison to its in vitro counterpart. The same observations can be made for several methods cited in this article such as embryo desiccation or the shoot apical meristem methods.

At present, the specific reasons slowing a wider adoption of these in planta approaches in the scientific community remain elusive as many of these techniques were demonstrated to be efficient in a large number of species. On the whole, this paper tried to review as many sources as possible, including those hard-to-access research articles, to build a compendium of references and provide the most accurate picture of a field that is rapidly evolving. In their reviews, Kaur and Devi [5] suggested that the field of in planta research is still in its early stages of development. While we understand the reasons underlying this standpoint, we would like to add some nuances. *In planta* research has always been at the core of transformation research since its beginning, and the floral dip approach in *Arabidopsis* is still a major propeller for the development of plant molecular biology. Several approaches are now in their mature phases, especially for dicots, with standardized protocols for a large number of species. In the longer term, many strategies targeting dicots, such as the tissue culture-independent de novo shoot induction method, clearly have the potential to become a mainstay of plant transformation. On the other hand, in planta techniques for monocots are less advanced, less diversified, and often more challenging to operate. Nonetheless, several approaches (e.g. pollen-tube pathway and shoot apical meristem injury methods) have already demonstrated their potential and are used regularly by several labs across the world. In conclusion, the in planta transformation concept offers important contributions to plant biotechnology by offering an alternative to traditional transformation/regeneration techniques and will surely become an increasingly important player in the field of plant transformation in the future.

## Online compendium

To further strengthen the content of this compendium, we solicit the support and help of the community to add additional references to the online version of this document available at https://github.com/Inplanta/In_planta_transformation. To do so, people can send their annotated references to the ‘’Issue’’ section of the GitHub page under the following format: (i) Family; (ii) Genera; (iii) Species; (iv) Common name; (v) Type of explant; (vi) Method; (vii) Notes; and (viii) Complete reference. The references should be in an Excel format and need to be submitted along with the original document. The compendium was built to limit in-text citations and provide a user-friendly versatile document to group and annotate in planta references. Overall, video footage showing specific methodological aspects is considered to be particularly helpful for the understanding and replicability of the techniques. To maximize the understanding of this paper, readers are invited to consult the compendium as they are reading.

## Electronic supplementary material

Below is the link to the electronic supplementary material.

**Supplementary Material 1: Table S1.** Compendium of in planta transformation strategies

**Supplementary Material 2: Table S2.** Benefits and limitations of different in planta transformation strategies

## Data Availability

A free, online, and up-to-date version of the in planta compendium is available at https://github.com/Inplanta/In_planta_transformation.

## Footnotes

1 The transformation rates provided in the article have been often calculated using different methods and cannot, in a large number of cases, be directly used for comparison.
