# Correction: A comprehensive review of in planta stable transformation strategies

**DOI:** 10.1186/s13007-024-01282-4

**Published:** 2024-10-15

**Authors:** Jérôme Bélanger, Tanya Rose Copley, Valerio Hoyos-Villegas, Jean-Benoit Charron, Louise O’Donoughue

**Affiliations:** 1 Centre de recherche sur les grains (CÉROM) Inc., 740 Chemin Trudeau, St-Mathieu-de-Beloeil, Québec, J3G 0E2 Canada; 2 Department of Plant Science, McGill University, 21111 Lakeshore Road, St-Mathieu-de-Beloeil, Montréal, Québec, H9X 3V9 Canada

**Correction: Plant Methods (2024) 20:79**

10.1186/s13007-024-01200-8

The wrong Supplementary file was originally published with this article; it has now been replaced with the correct file. Replaced the current Supplementary Table 2 with the revised Table S2 file.

The original article has been corrected.

## Electronic Supplementary Material

Below is the link to the electronic supplementary material.

Supplementary Material 1

Supplementary Material 2

