# Supplementary material for: A comprehensive review of in planta stable transformation strategies
Source: Plant Methods. 2024 May 31;20:79. doi: 10.1186/s13007-024-01200-8 (PMC11140912; doi:10.1186/s13007-024-01200-8)
Supplement: Supplementary file 2 — Supplementary Material 2: Table S2. Benefits and limitations of different in planta transformation strategies [file 13007_2024_1200_MOESM2_ESM.docx]

**Table S2. Benefits and limitations of different *in planta* transformation strategies**

| **Explant** | **Technique** | **Benefits** | **Limitations** | **References** |
| --- | --- | --- | --- | --- |
| Gamete (Ovule) | Vacuum-infiltration of inflorescence | - Simple - Higher efficiency than the floral dip method - High-throughput - No somaclonal variation | - Lower speed and more preparation than floral dip - Limited mostly to Brassicaceae - Hemizygous offspring (not always) | (1,2) |
|  |  |  |  |  |
| Gamete (Ovule) | Floral dip | - Simple - High-throughput - No somaclonal variation | - Limited mostly to Brassicaceae - Hemizygous offspring | (1,2) |
|  |  |  |  |  |
| Gamete (Ovule) | Floral bud injection / Floral bud painting / Floral bud spray | - Applicable to some species not amenable to the floral dip method - No somaclonal variation | - Low speed - Few protocols available - Technically challenging in comparison to floral dip - Hemizygous offspring (not always) | (1,2) |
|  |  |  |  |  |
| Gamete (Pollen) | Pollen transformation | - Pollen grains are easily isolated and occur in a large number - No somaclonal variation | - Few protocols available - Poor reproducibility - No standardized methods available - Challenging | (3,4) |
|  |  |  |  |  |
| Embryo | Pollen-Tube Pathway | - A large range of protocols - Demonstrated in a large number of species - No somaclonal variation | - Transformation efficiencies generally low - Hard-to-access literature - Inconsistent results in the past - Species-specific considerations - Higher level of technical requirements than some other methods | (5) |
|  |  |  |  |  |
| Embryo | Ovary-Drip | - Higher transformation efficiencies than pollen-tube pathway - No somaclonal variation | - Validated only in soybean and maize (requires broader validation) - Requires a high level of technical skills because it can wound the ovaries | (5) |
|  |  |  |  |  |
| Embryo | Pollen-Tube Agroinjection | - Simple - High rates of transformation - No somaclonal variation | - Validated only in peanut (requires broader validation) - Requires a higher level of technical skills than some other methods | (6) |
|  |  |  |  |  |
| Embryo | Ovary Injection Transformation | - Simple - High transformation efficiency in some species - No somaclonal variation | - Few protocols available - Confirmed only in a few species (require broader validation) - Possibly challenging to standardize | (1,7,8) |
|  |  |  |  |  |
| Embryo | Infection of Pre-Imbibed Embryos with Agrobacterium | - Simple and easily reproducible - Can be easily reproduced - High-throughput - No somaclonal variation | - Low transformation efficiency in most species - Requires validation in a broader range of species | (9–11) |
|  |  |  |  |  |
| Embryo | Agro-Imbibition | - Simple - High transformation efficiency according to the protocol - Low technical requirements - High-throughput - No somaclonal variation | - Patented - Requires validation from other research groups | (12) |
|  |  |  |  |  |
| Embryo | Imbibition of Desiccated Embryos | - Simple - Easy to reproduce - Low technical requirements - High-throughput - No somaclonal variation | - Few protocols available - Needs broader validation in a larger number of species | (13) |
|  |  |  |  |  |
| Shoot apical and adventitious meristems | Shoot apical meristem injury under *in vivo* conditions | - Simple - Universal (can be performed in all species) - Easy to reproduce - No somaclonal variation | - Low transformation efficiency - Chimeric (screening in T_1_ generation) | (14–16) |
|  |  |  |  |  |
| Shoot apical and adventitious meristems | Plumular Meristem Strategy | - Simple - High transformation efficiency - Easy to reproduce - No somaclonal variation | - Validated only in chickpea and pigeon pea (requires broader validation) - Not possible in monocots - *In vitro* conditions at the beginning limit broad adoption - Chimeric (screening in T_1_ generation) | (17–19) |
|  |  |  |  |  |
| Shoot apical and adventitious meristems | Direct Delivery | - High transformation efficiency - No somaclonal variation | - Inconsistent results in some species - Requires broader validation with a larger number of species and genotypes - Can generate plants with abnormal morphological features | (20–23) |
|  |  |  |  |  |
| Shoot apical and adventitious meristems | Nodal agroinjection | - Simple - High transformation efficiency - Easy to reproduce - No somaclonal variation | - Validated only in peanut (requires broader validation) - Chimeric (screening in T_1_ generation) | (24,25) |
|  |  |  |  |  |
| Shoot apical and adventitious meristems (propagule) | Cut-dip-budding | - Simple - Easy to reproduce - High transformation efficiency - No somaclonal variation | - Regeneration can be slow - Chimerism might be a potential problem - Only possible in plants that are vegetatively propagated | (26) |
|  |  |  |  |  |
| Shoot apical and adventitious meristems (propagule) | Regenerative Activity-dependent in Planta Injection Delivery | - Simple - Easy to reproduce - High transformation efficiency - No somaclonal variation | - Regeneration can be slow - Chimerism might be a potential problem - Only possible in plants that are vegetatively propagated | (27) |
|  |  |  |  |  |
| Shoot apical and adventitious meristems (propagule) | General methods of propagule transformation | - Simple - Easy to reproduce - High transformation efficiency - No somaclonal variation | - Regeneration can be slow - Chimerism might be a potential problem - Only possible in plants that are vegetatively propagated | (28–32) |
|  |  |  |  |  |
| Vegetative tissues | Callus-based transformation system | - Relatively simple - Can be used for tree transformation (e.g. poplar, longan, citrus) - No chimerism | - Possible somaclonal variation - Possible influence of the genotype on the regeneration capacity - Longer regeneration time - Need broader validation and standardized protocol in a larger number of species - Possible chimerism due to direct regeneration (33–35) - Not suitable for monocots | (33–37) |
|  |  |  |  |  |
| Embryonic axis and shoot apical meristems | *In vitro* direct organogenesis | - Relatively simple in comparison to the indirect pathway under *in vitro* conditions - Rapid regeneration - Can be applied to a large number of species (suitable for monocots and dicots) | - *In vitro* conditions decrease the overall efficiency - Protocols might require additional validation and optimization depending on the species | (38–40) |
|  |  |  |  |  |
| Novel | Grafting-mediated transformation | - Relatively high-throughput - No somaclonal variation | - Validated only in *Arabidopsis* and Brassica rapa (requires broader validation) - Possible influence of the genotype - Not suitable for monocots | (41) |
|  |  |  |  |  |
| Novel | Viral-based vectors | No somaclonal variation | - Challenging and requiring broader validation - Limited host range - Limited cargo capacity - Most viral-based method requires *in vitro* conditions, except those using an FT-based cassette   Limited vector mobility | (42–45) |

**References**

1. Jan SA, Shinwari ZK, Shah SH, Shahzad A, Zia MA, Ahmad N. In-planta transformation: recent advances. Rom Biotechnol Lett. 2016;21(1):11085–91.

2. Bent A. Arabidopsis thaliana floral dip transformation method. Methods Mol Biol. 2006;343:87–103.

3. Eapen S. Pollen grains as a target for introduction of foreign genes into plants: An assessment. Physiol Mol Biol Plants. 2011;17(1):1–8.

4. Harwood WA, Chen DF, Creissen GP. Transformation of Pollen and microspores A review. 1996;2:53–71.

5. Ali A, Bang SW, Chung SM, Staub JE. Plant Transformation via Pollen Tube-Mediated Gene Transfer. Plant Mol Biol Report. 2015;33(3):742–7.

6. Zhou M, Luo J, Xiao D, Wang A, He L, Zhan J. An efficient method for the production of transgenic peanut plants by pollen tube transformation mediated by Agrobacterium tumefaciens. Plant Cell Tissue Organ Cult. 2023;152(1):207–14.

7. Bahari Z, Sazegari S, Niazi A, Afsharifar A. The application of an agrobacterium-mediated in planta transformation system in a catharanthus Roseus medicinal plant. Czech J Genet Plant Breed. 2020;56(1):34–41.

8. Zia M, Arshad W, Bibi Y, Nisa S, Chaudhary MF. Does Agro-injection to soybean pods transform embryos? Plant Omics. 2011;4(7):384–90.

9. Graves ACF, Goldman SL. The transformation of Zea mays seedlings with Agrobacterium tumefaciens. Plant Mol Biol. 1986;7(1):43–50.

10. Chee P, Goldman S, Graves A, Slightom J. Agrobacterium Mediated Transformation of Germinating Plant Seeds (US Patent US5169770A) [Internet]. EP: UPJOHN CO; 1990. Available from: https://lens.org/117-765-919-543-113

11. Chee PP, Fober KA, Slightom JL. Transformation of Soybean ( Glycine max ) by Infecting Germinating Seeds with Agrobacterium tumefaciens. Plant Physiol. 1989;91(3):1212–8.

12. Kharb P, Chaudhary R, Tuteja N, Kaushik P. A Genotype-Independent, Simple, Effective and Efficient in Planta Agrobacterium-Mediated Genetic Transformation Protocol. Methods Protoc. 2022;5(5):69.

13. Arias D, Mckersie B, Taylor J. In planta transformation by embryo imbibition of Agrobacterium (Patent US 31478001) [Internet]. CA: BASF PLANT SCIENCE GMBH OP - US 31478001 P OP - US 0227164 W; 2003. Available from: https://lens.org/144-522-898-736-153

14. Kesiraju K, Sreevathsa R. Apical meristem-targeted in planta transformation strategy: an overview on its utility in crop improvement. Agri Res Technol Open Access J. 2017;8(555734):10–19080.

15. Sankara Rao K, Rohini VK. A novel in planta approach to gene transfer for legumes. In: Handbook of New Technologies for Genetic Improvement of Legumes. CRC Press; 2008. p. 273–86.

16. Zlobin NE, Lebedeva M V., Taranov V V. CRISPR/Cas9 genome editing through in planta transformation. Crit Rev Biotechnol [Internet]. 2020;40(2):153–68. Available from: https://doi.org/10.1080/07388551.2019.1709795

17. Ganguly S, Ghosh G, Ghosh S, Purohit A, Chaudhuri RK, Das S, et al. Plumular meristem transformation system for chickpea: an efficient method to overcome recalcitrant tissue culture responses. Plant Cell Tissue Organ Cult [Internet]. 2020;142(3):493–504. Available from: https://doi.org/10.1007/s11240-020-01873-8

18. Ganguly S, Purohit A, Chaudhuri RK, Das S, Chakraborti D. Embryonic explant and plumular meristem transformation methods for development of transgenic pigeon pea. In: Methods in Molecular Biology. Springer; 2020. p. 317–33.

19. Ganguly S, Ghosh G, Purohit A, Kundu Chaudhuri R, Chakraborti D. Development of transgenic pigeonpea using high throughput plumular meristem transformation method. Plant Cell Tissue Organ Cult [Internet]. 2018;135(1):73–83. Available from: http://dx.doi.org/10.1007/s11240-018-1444-3

20. Nasti RA, Voytas DF. Attaining the promise of plant gene editing at scale. Proc Natl Acad Sci U S A. 2021;118(22):1–6.

21. Cody JP, Maher MF, Nasti RA, Starker CG, James C, Chamness JC, et al. Direct delivery and fast-treated Agrobacterium co-culture (Fast-TrACC) plant transformation methods for Nicotiana benthamiana. Nat Protoc. 2023;18(1):81–107.

22. Lian Z, Nguyen CD, Liu L, Wang G, Chen J, Wang S, et al. Application of developmental regulators to improve in planta or in vitro transformation in plants. Plant Biotechnol J. 2022;20(8):1622–35.

23. Nasti RA, Zinselmeier MH, Vollbrecht M, Maher MF, Voytas DF. Fast-TrACC: A Rapid Method for Delivering and Testing Gene Editing Reagents in Somatic Plant Cells. Front Genome Ed. 2021;2(January):1–9.

24. Wang C, Wang X, Tang Y, Wu Q, Li G, Song G, et al. Transforming peanut (Arachis hypogaea L.): a simple in planta method. Res Crop. 2013;14(3):850–4.

25. Han HW, Yu ST, Wang ZW, Yang Z, Jiang CJ, Wang XZ, et al. In planta genetic transformation to produce CRISPRed high-oleic peanut. Plant Growth Regul [Internet]. 2023;101(2):443–51. Available from: https://doi.org/10.1007/s10725-023-01031-y

26. Cao X, Xie H, Song M, Lu J, Ma P, Huang B, et al. Cut–dip–budding delivery system enables genetic modifications in plants without tissue culture. Innovation [Internet]. 2023 Jan 30;4(1):100345. Available from: https://doi.org/10.1016/j.xinn.2022.100345

27. Mei G, Chen A, Wang Y, Li S, Wu M, Liu X, et al. A simple and efficient in planta transformation method based on the active regeneration capacity of plants. bioRxiv [Internet]. 2023;2023.01.02.522521. Available from: https://doi.org/10.1101/2023.01.02.522521

28. Ramasamy S, Chelliah A, R RM, Mohanraj MS, Velusamy B. Agrobacterium-mediated In planta Transformation of Hill Banana for Developing Resistance against Banana Bunchy Top Virus. Madras Agric J. 2011;98(December):374–8.

29. Xu N, ming Man J, Luo R. A non-tissue culture dependent genetic transformation of Marchantia polymorphya L. (Pre-print). ResearchSquare [Internet]. 2023; Available from: https://www.researchsquare.com/article/rs-2556225/v1

30. Jung Y, Rhee Y, Auh CK, Shim H, Choi JJ, Kwon ST, et al. Production off recombinant single chain antibodies (scFv) in vegetatively reproductive Kalanchoe pinnata by in planta transformation. Plant Cell Rep. 2009;28(10):1593–602.

31. Mayavan S, Subramanyam K, Jaganath B, Sathish D, Manickavasagam M, Ganapathi A. Agrobacterium-mediated in planta genetic transformation of sugarcane setts. Plant Cell Rep. 2015 Oct 22;34(10):1835–48.

32. Seol E, Jung Y, Lee J, Cho C, Kim T, Rhee Y, et al. In planta transformation of Notocactus scopa cv. Soonjung by Agrobacterium tumefaciens. Plant Cell Rep. 2008 Jul;27(7):1197–206.

33. Rizwan HM, Yang Q, Yousef AF, Zhang X, Sharif Y, Kaijie J, et al. Establishment of a novel and efficient agrobacterium-mediated in planta transformation system for passion fruit (Passiflora edulis). Plants. 2021;10(11):2459.

34. Chen YK, Cheng CZ, Zhang ZH, Lai Z. Establishment of a rapid transgenic method on longan seedling. Chin J Appl Env Biol. 2020;26(6):1540–5.

35. Yukun C, Chunzen C, Zhongxiong L, Yuling L, Shengcai L, Zihao Z. Rapid transgenic method for longan (Patent CN 105505991 A) [Internet]. CN: UNIV FUJIAN AGRICULTURE & FORESTRY OP - CN 201610059720 A; 2016. Available from: https://lens.org/029-715-019-242-790

36. Zhang Y, Zhang D, Zhong Y, Chang X, Hu M, Cheng C. A simple and efficient in planta transformation method for pommelo (Citrus maxima) using Agrobacterium tumefaciens. Sci Hortic (Amsterdam) [Internet]. 2017;214:174–9. Available from: http://dx.doi.org/10.1016/j.scienta.2016.11.033

37. Mily R, Neelima S, Anne B, Moran F. Generation of Heritably Gene-Edited Plants Without Tissue Culture (Patent US 201862727431) [Internet]. Vol. 1. WO, United States of America: UNIV CALIFORNIA OP - US 201862727431 P; 2020. Available from: https://lens.org/011-524-499-432-378

38. Paes de Melo B, Lourenço-Tessutti IT, Morgante CV, Santos NC, Pinheiro LB, de Jesus Lins CB, et al. Soybean Embryonic Axis Transformation: Combining Biolistic and Agrobacterium-Mediated Protocols to Overcome Typical Complications of In Vitro Plant Regeneration. Front Plant Sci. 2020;11(August):1–14.

39. Ribeiro TP, Lourenço-Tessutti IT, de Melo BP, Morgante CV, Filho AS, Lins CBJ, et al. Improved cotton transformation protocol mediated by Agrobacterium and biolistic combined-methods. Planta [Internet]. 2021;254(2):1–14. Available from: https://doi.org/10.1007/s00425-021-03666-5

40. Cho HJ, Moy Y, Rudnick NA, Klein TM, Yin J, Bolar J, et al. Development of an efficient marker-free soybean transformation method using the novel bacterium Ochrobactrum haywardense H1. Plant Biotechnol J. 2022;20(5):977–90.

41. Yang L, Machin F, Wang S, Saplaoura E, Kragler F. Heritable transgene-free genome editing in plants by grafting of wild-type shoots to transgenic donor rootstocks. Nat Biotechnol. 2023;41:958–967.

42. Oh Y, Kim H, Kim SG. Virus-induced plant genome editing. Curr Opin Plant Biol [Internet]. 2021;60:101992. Available from: https://doi.org/10.1016/j.pbi.2020.101992

43. Shan-E-Ali Zaidi S, Mansoor S. Viral vectors for plant genome engineering. Front Plant Sci. 2017;8(April):2012–7.

44. Zhang C, Liu S, Li X, Zhang R, Li J. Virus-Induced Gene Editing and Its Applications in Plants. Int J Mol Sci. 2022;23(18):10202.

45. Mahmood MA, Naqvi RZ, Rahman SU, Amin I, Mansoor S. Plant Virus-Derived Vectors for Plant Genome Engineering. Viruses. 2023;15(2):1–20.
